# *Lactobacillus-*derived protoporphyrin IX and SCFAs regulate the fiber size via glucose metabolism in the skeletal muscle of chickens

**DOI:** 10.1128/msystems.00214-24

**Published:** 2024-05-23

**Authors:** Liyuan Cai, Xinkai Wang, Xiaoyan Zhu, Yunzheng Xu, Wenxia Qin, Jing Ren, Qin Jiang, Xianghua Yan

**Affiliations:** 1 National Key Laboratory of Agricultural Microbiology, Frontiers Science Center for Animal Breeding and Sustainable Production, Hubei Hongshan Laboratory, College of Animal Sciences and Technology, Huazhong Agricultural University, Wuhan, Hubei, China; 2 Shandong Teamgene Technology Co. Ltd., Zibo, Shandong, China; The University of Maine, Orono, Maine, USA

**Keywords:** chicken, *Lactobacillus*, skeletal muscle, glucose metabolism, fiber size

## Abstract

The gut microbiota contributes to skeletal muscle energy metabolism and is an indirect factor affecting meat quality. However, the role of specific gut microbes in energy metabolism and fiber size of skeletal muscle in chickens remains largely unknown. In this study, we first performed cecal microbiota transplantation from Chinese indigenous Jingyuan chickens (JY) to Arbor Acres chickens (AA), to determine the effects of microbiota on skeletal muscle fiber and energy metabolism. Then, we used metagenomics, gas chromatography, and metabolomics analysis to identify functional microbes. Finally, we validated the role of these functional microbes in regulating the fiber size via glucose metabolism in the skeletal muscle of chickens through feeding experiments. The results showed that the skeletal muscle characteristics of AA after microbiota transplantation tended to be consistent with that of JY, as the fiber diameter was significantly increased, and glucose metabolism level was significantly enhanced in the pectoralis muscle. *L. plantarum*, *L. ingluviei*, *L. salivarius,* and their mixture could increase the production of the microbial metabolites protoporphyrin IX and short-chain fatty acids, therefore increasing the expression levels of genes related to the oxidative fiber type (*MyHC SM* and *MyHC FRM*), mitochondrial function (*Tfam* and *CoxVa*), and glucose metabolism (*PFK*, *PK*, *PDH*, *IDH*, and *SDH*), thereby increasing the fiber diameter and density. These three *Lactobacillus* species could be promising probiotics to improve the meat quality of chicken.

**IMPORTANCE:**

This study revealed that the *L. plantarum, L. ingluviei*, and *L. salivarius* could enhance the production of protoporphyrin IX and short-chain fatty acids in the cecum of chickens, improving glucose metabolism, and finally cause the increase in fiber diameter and density of skeletal muscle. These three microbes could be potential probiotic candidates to regulate glucose metabolism in skeletal muscle to improve the meat quality of chicken in broiler production.

## INTRODUCTION

Exploring effective strategies to improve meat quality is an urgent issue to be solved in broiler production to address the increasing requirements of consumers (1). Skeletal muscle, the material basis for meat, plays a critical role in insulin-stimulated glucose disposal and fatty acid oxidation, showing its fundamental importance in energy metabolism (2, 3). Variation in energy metabolism intensity can affect muscle fiber diameter and the proportion of oxidized and glycolytic fibers, affecting meat attributes such as tenderness, color, pH, juiciness, and flavor (2, 4). Therefore, regulating skeletal muscle energy metabolism is a potentially effective way to improve meat quality (5).

The gut microbiota is considered a type of “microbial organ” in animals that provides the host with some functions that are not encoded in the host genome but are essential for the physiological processes of the host and play important roles in nutrient utilization and energy metabolism (6). Many studies have focused on the role of the gut microbiota in type 2 diabetes, obesity, immune performance, gut metabolism, liver metabolism, and behaviors (7–11). However, few studies investigated the role of the gut microbiota in energy metabolism in skeletal muscle. Recent studies have reported a close relationship between certain skeletal muscle diseases, age-related muscular atrophy, and the gut microbiota in humans (12–14). Moreover, it has been reported that certain gut microbial taxa have relevant effects on enhancing energy metabolism in the skeletal muscle of the host by producing energetic compounds (12, 14, 15). In mice, growth, atrophy, and the metabolism of branched chain amino acids, fatty acids, and glucose in skeletal muscle are influenced by the gut microbiota (3). Compared with mammals, chickens have distinctly different characteristics in the physiological structure of the digestive tract and nutrient metabolism (16). However, the role of the gut microbiota in chicken skeletal muscle energy metabolism remains unexplored.

The aim of this study was to reveal the potential regulatory mechanisms of the gut microbiota on the fiber size and energy metabolism in the skeletal muscle of chickens.

We hypothesized that functional gut microbiota plays an important role in regulating fiber size by enhancing energy metabolism in the skeletal muscle of chickens. We employed cecal microbiota transplantation, metagenomics, metabonomics, gas chromatograph, and feeding experiments to validate functional microbes that regulate fiber size by enhancing glucose metabolism in the skeletal muscle of chickens.

## RESULTS

### Fiber size and energy metabolism in skeletal muscle were significantly different between JY and AA

**Fig 1:**
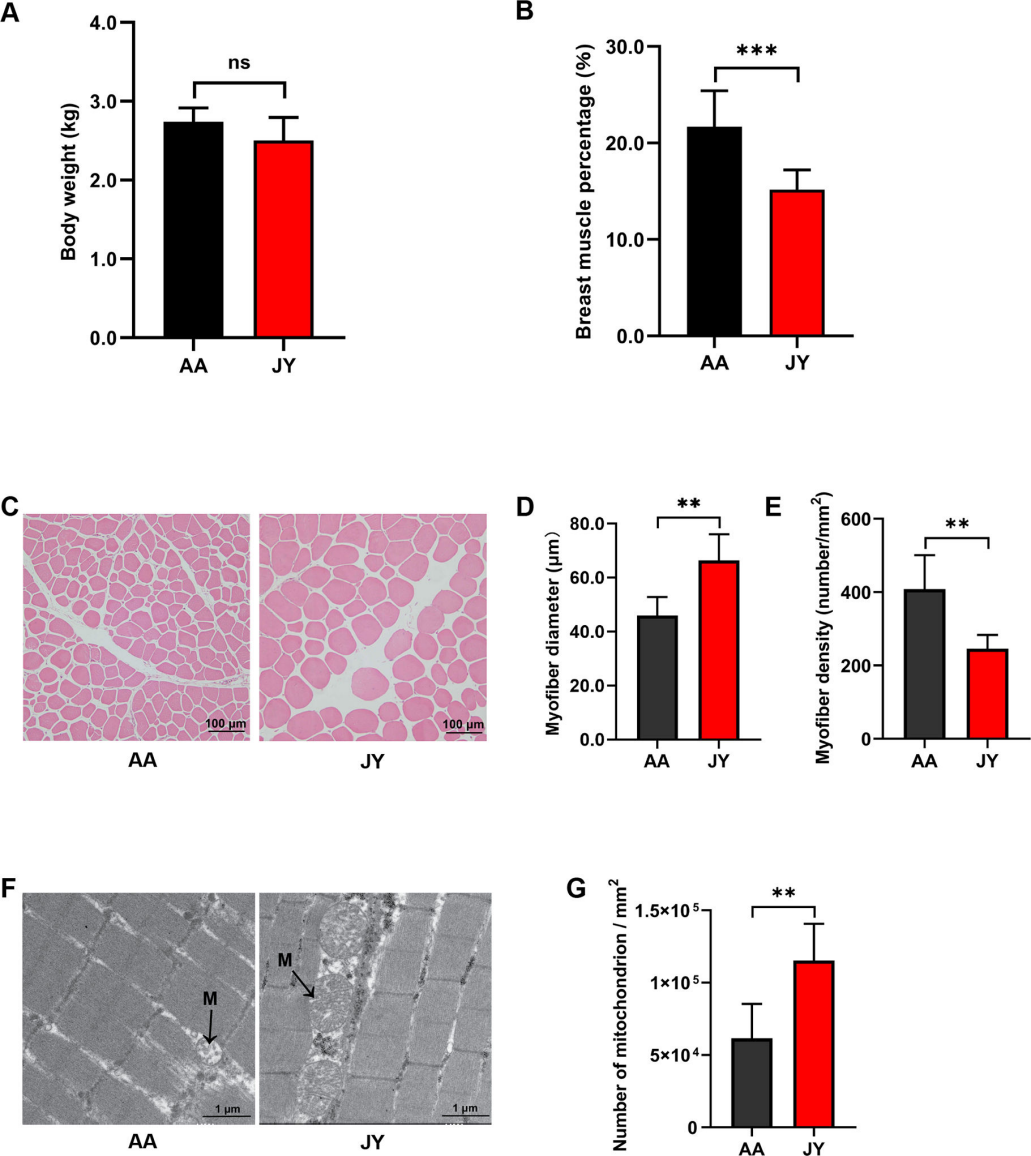
Body weight and skeletal muscle characteristics of AA and JY. (**A**) Body weight of AA and JY. (**B**) The breast muscle percentage of AA and JY. (**C**) HE staining (original magnification, ×100) shows the fiber size of the pectoralis muscle in AA and JY. (**D**) The pectoralis muscle fiber diameters and (**E**) densities in AA and JY. (**F**) Transmission electron micrograph (original magnification, ×5,000) of longitudinal sections of the pectoralis muscle in AA and JY. A mitochondrion is presented in the “M” site. (**G**) The number of mitochondria per square millimeter in the pectoralis muscle of AA and JY. The data were analyzed using a two-tailed Student’s *t test* and were considered statistically significant at *P < 0.05, **P < 0.01, and ***P < 0.001 and not significant (ns) when *P* > 0.05, between the indicated groups. The data are expressed as the mean ± SEM (*n* = 6 chickens).

There were no significant differences in body weight between the Arbor Acres (AA) and Jingyuan (JY) chickens (Fig. 1A), whereas the breast muscle percentage of JY was significantly lower than that in AA (*P* < 0.001; Fig. 1B). The fiber size of the pectoralis muscle was observed using hematoxylin-eosin (HE) staining (Fig. 1C). Compared to AA, the fiber diameter was significantly increased (*P* < 0.01; Fig. 1D), whereas the fiber density of the pectoralis muscle was significantly decreased in JY (*P* < 0.01; Fig. 1E). Moreover, the number of mitochondria in the pectoralis muscle was observed by transmission electron microscopy (TEM) (Fig. 1F). Compared to AA, the mitochondrial number per square millimeter was significantly increased (*P* < 0.01; Fig. 1G) in the pectoralis muscle of JY.

**Fig 2:**
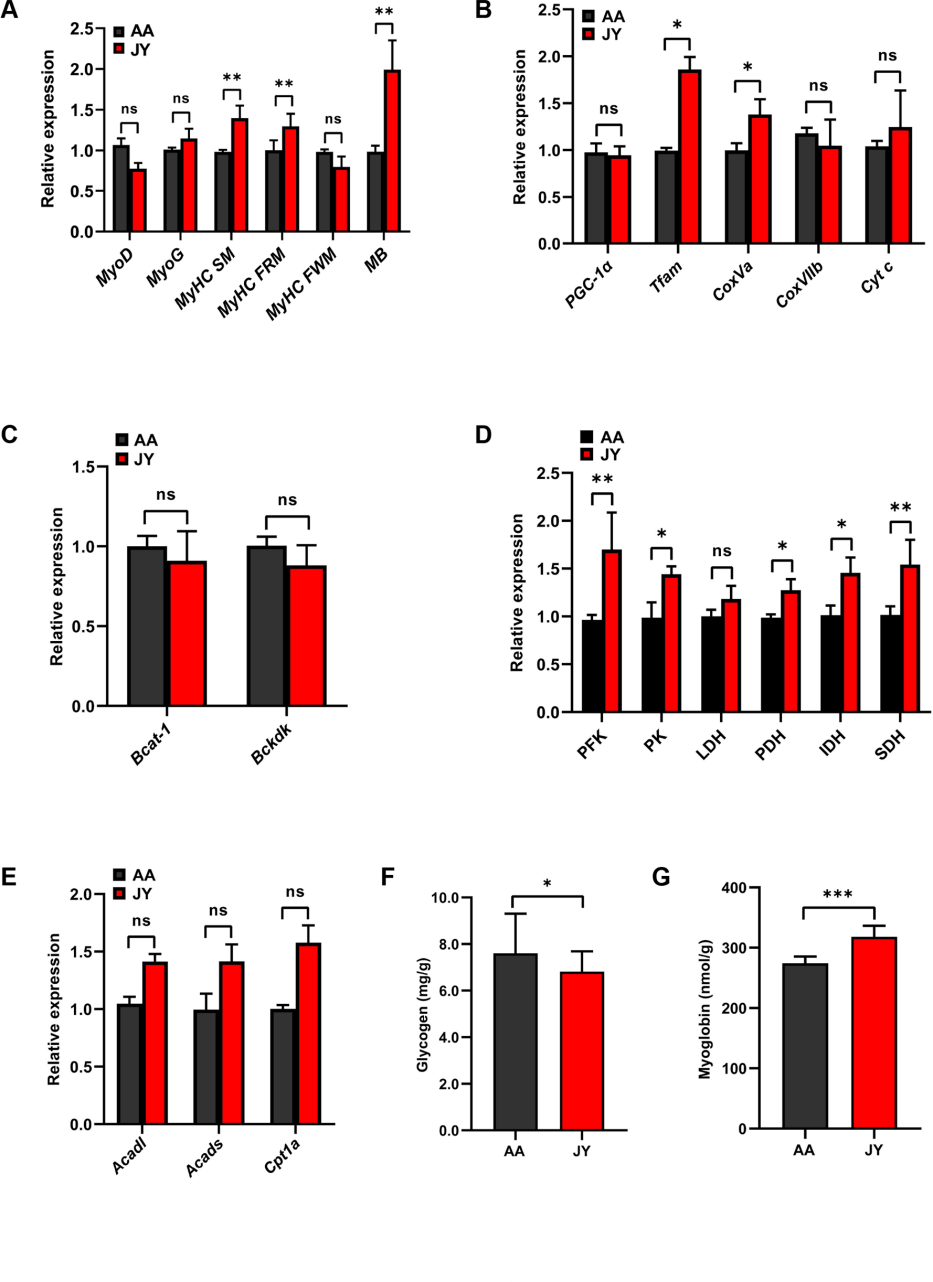
Gene expression in the skeletal muscle of AA and JY. (**A**) The expression of the skeletal muscle-specific transcription factors *MyoD* and *MyoD*; genes encoding myosin heavy chain (MyHC) isoforms, namely, *MyHC SM*, *MyHC FRM*, and *MyHC FWM*; and the myoglobin (*MB*) gene in the pectoralis muscle of AA and JY. (**B**) The expression of the *PGC-1*α, *Tfam*, *CoxVa*, *CoxVIIb*, and *Cyt c* genes related to mitochondrial function in the pectoralis muscle of AA and JY. (**C**) The expression of the *Bcat-1* and *Bckdk* genes involved in branched-chain amino acid catabolism in the pectoralis muscle of AA and JY. (**D**) The expression of the *PFK*, *PK*, *LDH*, *PDH*, and *SDH* genes involved in glucose metabolism in the pectoralis muscle of AA and JY. (**E**) The expression of the *Acadl*, *Acads*, and C*pt1a* genes involved in the fatty acid oxidation pathway in the pectoralis muscle of AA and JY. (**F**) The concentration of glycogen in the pectoralis muscle of AA and JY. (**G**) The concentration of myoglobin in the pectoralis muscle of AA and JY. The data were analyzed using a two-tailed Student’s *t test* and were considered statistically significant at *P < 0.05, **P < 0.01, and ***P < 0.001 and not significant (ns) when *P* > 0.05, between the indicated groups. The data are expressed as the mean ± SEM (*n* = 6 chickens).

To investigate the profiles of skeletal muscle growth and metabolism, gene expression related to skeletal muscle growth, differentiation, and the fiber type were investigated in the pectoralis muscle of chickens. The expression levels of the myosin heavy chain genes for slow-twitch (*MyHC SM*), red fast-twitch myofibers (*MyHC FRM*), and the myoglobin gene (*MB*) in the pectoralis muscle of JY were significantly increased compared to those of AA (*P* < 0.01; Fig. 2A). However, there were no differences in the expression levels of myogenic differentiation antigen (*MyoD*), myogenin (*MyoG*), and myosin heavy chain gene for white fast-twitch myofibers (*MyHC FWM*) in the pectoralis muscle between AA and JY (Fig. 2A). These results prompted us to further investigate the energy metabolism capacity of the pectoralis muscle. Therefore, gene expression levels related to mitochondrial function were investigated. Compared to AA, an increased expression of mitochondrial transcription factor A (*Tfam*) and cytochrome oxidase subunits of complex IV CoxVa genes was observed in the pectoralis muscle of JY (*P* < 0.05; Fig. 2B). There were no differences in the expression levels of peroxisome proliferator-activated receptor-γ-coactivator 1α (*PGC-1α*), cytochrome oxidase subunits of complex IV *CoxVIIb,* and *Cyt c* genes in the pectoralis muscle between JY and AA (Fig. 2B). Furthermore, the expression levels of genes related to branched-chain amino acid metabolism, glucose metabolism, and lipid metabolism were further investigated. The expression levels of phosphofructokinase (*PFK*), pyruvate kinase (*PK*), pyruvate dehydrogenase (*PDH*), isocitrate dehydrogenase (*IDH*), and succinate dehydrogenase (*SDH*) in the pectoralis muscle of JY were significantly increased compared with those of AA (*P* < 0.05; Fig. 2D). However, there were no differences in the expression levels of the branched-chain amino acid transaminase 1 (*Bcat1*), branched-chain keto acid dehydrogenase kinase (*Bckdk*), lactate dehydrogenase (*LDH*), long-chain acyl-coenzyme A dehydrogenase (*Acadl*), short-chain acyl-coenzyme A dehydrogenase (*Acads*), and carnitine palmitoyltransferase 1a (*Cpt1a*) genes in the pectoralis muscle between AA and JY (Fig. 2C through E). In addition, compared to AA, significantly decreased glycogen (*P* < 0.05; Fig. 2F) and significantly increased myoglobin concentrations were observed in the pectoralis muscle of JY (*P* < 0.001; Fig. 2G).

### Cecal microbiota transplantation induces a change in skeletal muscle characteristics in chickens

**Fig 3:**
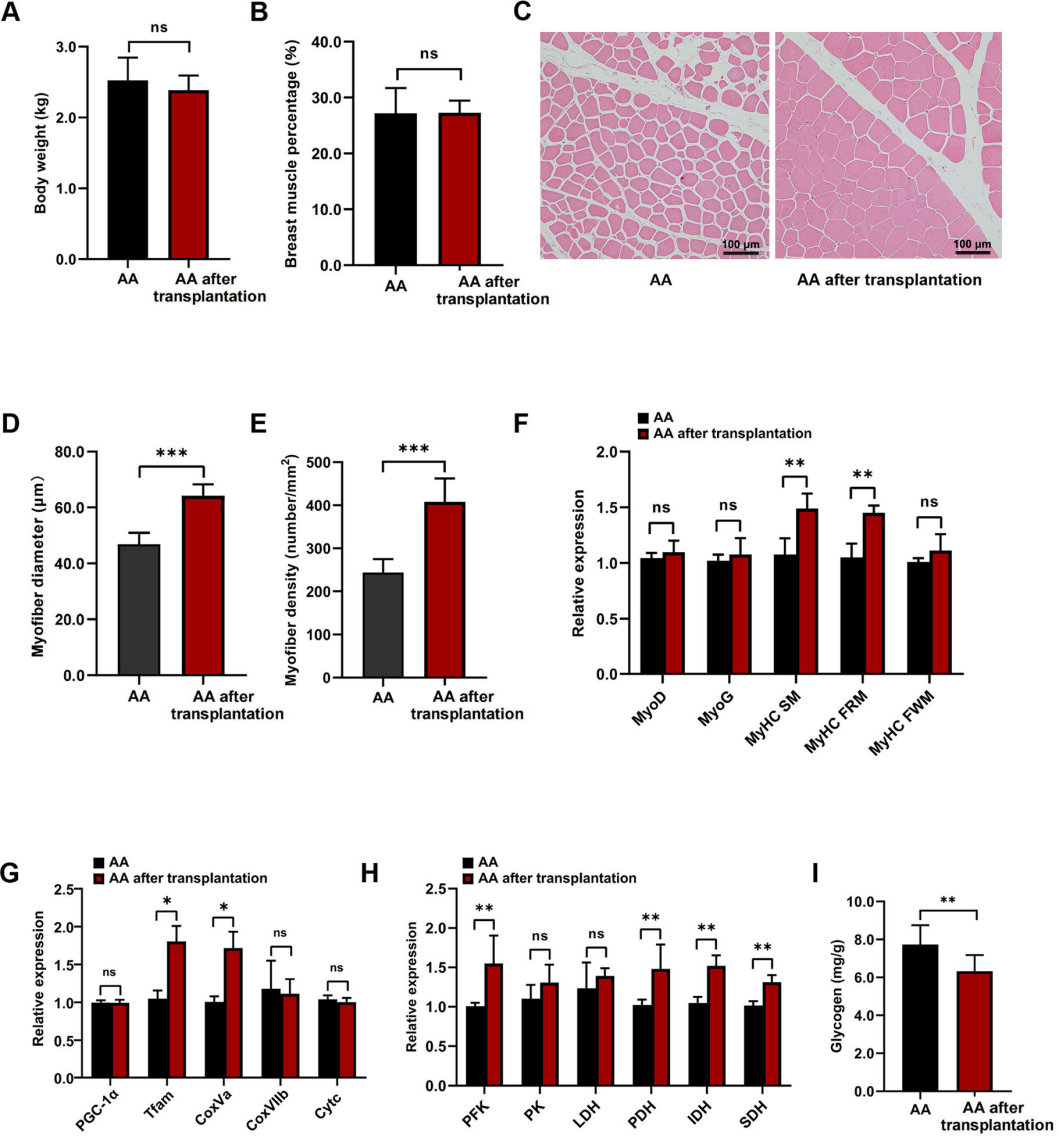
Body weight and pectoralis muscle characteristics of AA and AA after transplantation. (**A**) Body weight. (**B**) The breast muscle percentage of AA and AA after transplantation. (**C**) HE staining (original magnification, ×100) showing the fiber size of the pectoralis muscle in AA and AA after transplantation. (**D**) The pectoralis muscle fiber diameters and (**E**) densities of AA and AA after transplantation. (**F**) The expression of the skeletal muscle-specific transcription factors *MyoD* and *MyoD*; genes encoding myosin heavy chain (MyHC) isoforms, namely, *MyHC SM*, *MyHC FRM*, and *MyHC FWM*; and the myoglobin (*MB*) gene in the pectoralis muscle of AA and AA after transplantation. (**G**) The expression of the *PGC-1*α, *Tfam*, *CoxVa*, *CoxVIIb*, and *Cyt c* genes related to mitochondrial function in the pectoralis muscle of AA and AA after transplantation. (**H**) The expression of the *PFK*, *PK*, *LDH*, *PDH*, and *SDH* genes involved in glucose metabolism in the pectoralis muscle of AA and AA after transplantation. (**I**) The concentration of glycogen in the pectoralis muscle of AA and AA after transplantation. *P < 0.05, **P < 0.01, ***P < 0.001, and not significant (ns) when *P* > 0.05, between the indicated groups. The data are expressed as the mean ± SEM (*n* = 6 chickens).

To identify and validate the effects of the gut microbiota on the fiber size and glucose metabolism in the skeletal muscle of chickens, we transferred the cecal microbiota from JY to AA. The results showed that there were no differences in the body weight and breast muscle percentage ratio between the AA and AA after transplantation (*P* < 0.05, Fig. 3A and B). Compared with AA, the fiber diameter and density were significantly increased in the pectoralis muscle of AA after transplantation (*P* < 0.05; Fig. 3C through E). Moreover, the glucose metabolism level was enhanced in the pectoralis muscle after the microbiota transplantation. Compared to AA, there were increased expression levels of the *MyHC SM*, *MyHC FRM*, *Tfam*, *CoxVa*, *PFK*, *PK*, *PDH*, *IDH*, and *SDH* genes in the pectoralis muscle of the AA after transplantation (*P* < 0.05; Fig. 3F through H). However, there were no differences in the expression levels of *MyoD*, *MyoG*, *MyHC FWM*, *PGC-1*α, *CoxVIIb*, *Cyt c*, and *LDH* genes in the pectoralis muscle between AA and the AA after transplantation (Fig. 3F through H). In addition, compared to AA, a significantly decreased glycogen concentration was observed in the pectoralis muscle of the AA after transplantation (*P* < 0.01; Fig. 3I). These results suggest that the gut microbiota could influence the fiber size and glucose metabolism of skeletal muscle in chickens.

### Profiles of the cecal microbial metagenome of chickens

The metagenome sequencing generated a total of 164,753,615,700 reads, with 13,729,467,975 ± 427,140,926 reads (mean ± SEM) per sample (Table S1). After quality control (QC) and the removal of host genes, a total of 135,042,737,100 reads were retained, with 11,253,561,425 ± 593,551,246 per sample (Table S1). After *de novo* assembly, a total of 5,956,565 contigs were generated (N50 length of 1,678 ± 65 bp), with 496,380 ± 42,966 per sample (Table S1). The cecal microbial metagenome consisted of 96.41% bacteria (5,742,724 contigs), 3.10% archaea (184,654 contigs), and 0.49% viruses (29,187 contigs). There were no significant differences in bacteria, archaea, and viruses between the two groups at the kingdom level (Fig. S1A).

### AA after transplantation presents a distinct composition of gut microbiota compared to AA

The dominant microbial phyla included Bacteroidetes (53.00% ± 4.00%), Firmicutes (22.02% ± 3.32%), and Proteobacteria (20.86% ± 3.39%). The dominant microbial genera included *Bacteroides* (49.50% ± 4.234%), *Helicobacter* (16.76% ± 3.87%), *Lactobacillus* (4.96% ± 1.49%), and *Subdoligranulum* (4.67% ± 1.39%). The dominant microbial species was *Bacteroides barnesiae* (29.70% ± 5.47%), followed by *Helicobacter pullorum* (17.01% ± 3.90%), *Bacteroides coprocola* (9.01% ± 4.54%), *Bacteroides salanitronis* (5.12% ± 1.61%), and *Subdoligranulum* unclassified (4.79% ± 1.43%).

**Fig 4:**
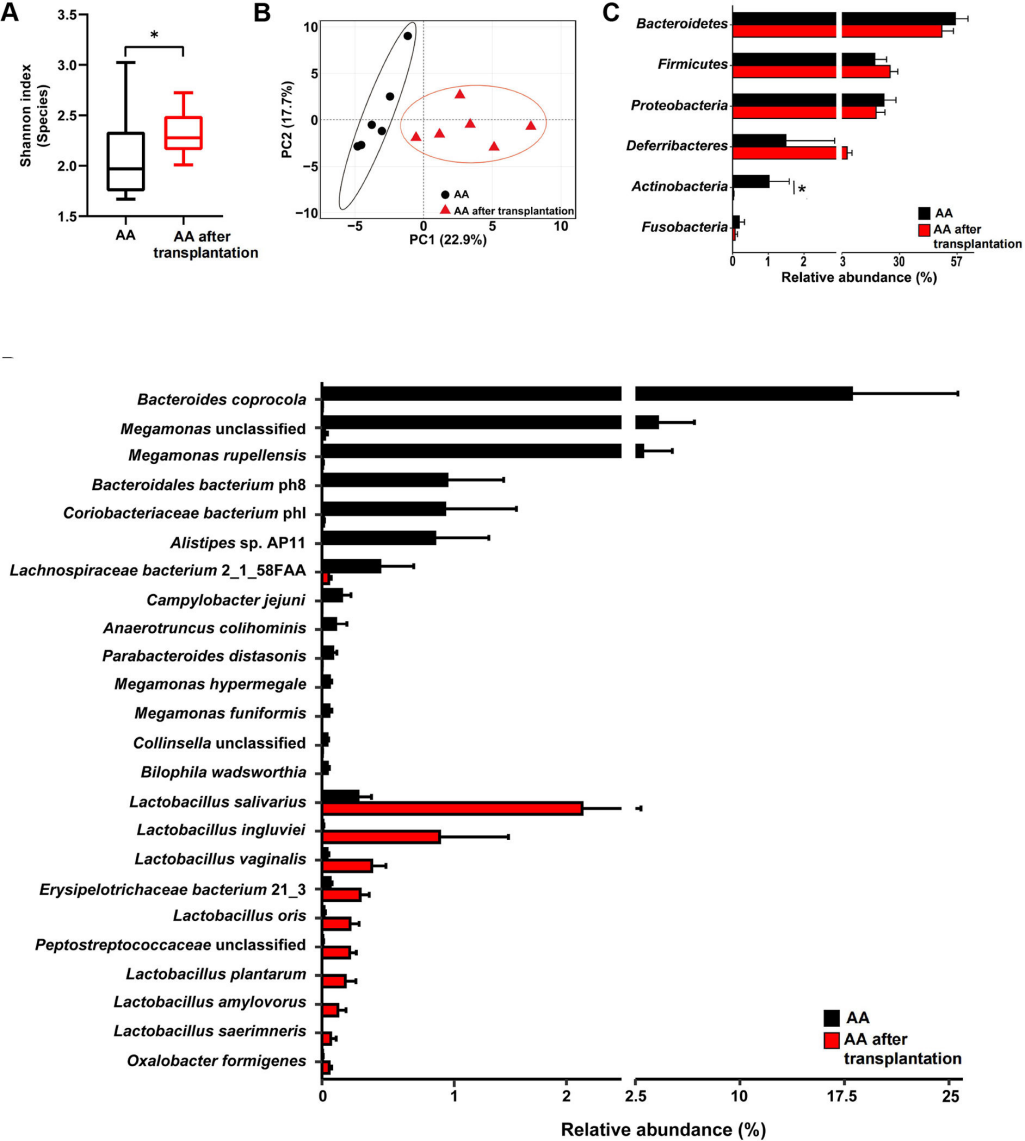
Profiles of cecal microbiome composition in AA and AA after transplantation. (**A**) Comparison of the microbial α-diversity (Shannon index) at the microbial species level between AA and AA after transplantation. (**B**) Microbial compositional profiles in the cecum of AA and AA after transplantation based on species visualized by a principal component analysis. (**C**) Microbial composition at the phylum level in the cecum of AA and AA after transplantation. All microbial phyla are displayed. (**D**) Differential microbial species in the cecum between AA and AA after transplantation. AA received sterilized PBS, and the AA after transplantation received 108 CFU/mL cecal microbial suspension. Only microbiota with *P* < 0.05 are displayed. The data are the means ± SEM (*n* = 6 chickens).

The Shannon index at the microbial species level was compared between AA and AA after transplantation, and this index of the latter was significantly higher than that in the former (*P* < 0.05; Fig. 4A). The principal component analysis (PCA) showed separations between AA and AA after transplantation based on the bacterial species (Fig. 4B). At the phylum level, the relative abundance of Actinobacteria was significantly higher in the cecum of AA (*P* < 0.05; Fig. 4C). At the genus level, the relative abundances of *Subdoligranulum, Leuconostoc, Peptostreptococcaceae* unclassified, and *Oxalobacter* were significantly higher in the cecum of AA after transplantation (*P* < 0.05; Fig. S1B) than that of AA, whereas *Bacteroidales* unclassified, *Megamonas*, *Coriobacteriaceae* unclassified, and the other four microbial genera were significantly higher in the cecum of AA (*P* < 0.05; Fig. S1B). At the species level, *Lactobacillus salivarius*, *Lactobacillus ingluviei*, *Lactobacillus vaginalis*, and 7 other microbial genera exhibited significantly higher relative abundances in the cecum of AA after transplantation, whereas *Bacteroides coprocola*, *Megamonas* unclassified, *Megamonas rupellensis*, and 11 other microbial genera exhibited significant enrichment in the cecum of AA (*P* < 0.05; Fig. 4D).

### AA after transplantation presents a distinct function of the gut microbiota compared to AA

The functions of the cecal microbiota were determined using KEGG profiles. In these profiles, 173 endogenous level-3 pathways were considered cecal microbial metabolic pathways (Table S2). These pathways belonged to four level-1 categories, including “Metabolism” (58.56% ± 1.14%), “Genetic information processing” (24.35% ± 0.65%), “Environmental information processing” (11.38% ± 0.34%), and “Cellular processes” (5.71% ± 0.22%). In total, 21 pathways belonged to KEGG level-2 categories, with “Carbohydrate metabolism” (11.16% ± 0.29%), “Translation” (9.87% ± 0.36%), and “Replication and repair” (9.17% ± 0.17%) being the most abundant pathways.

**Fig 5:**
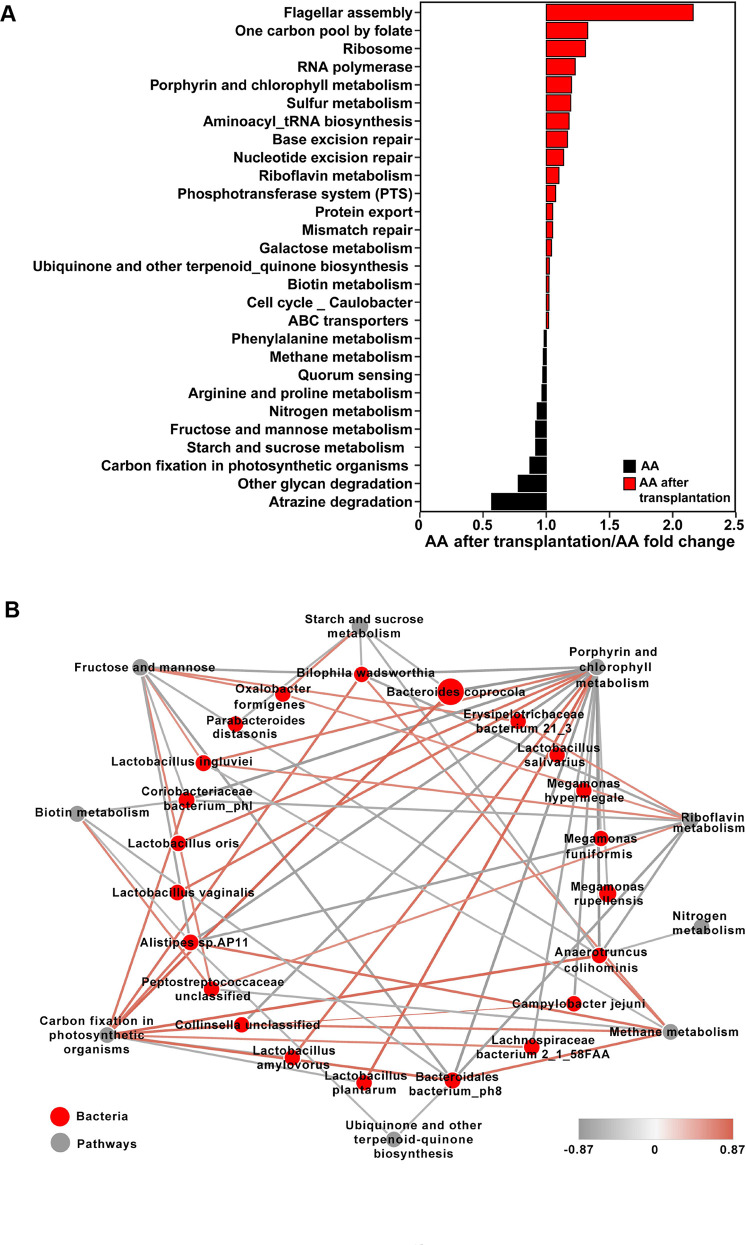
Functions of the cecal microbiota in AA and AA after transplantation. (**A**) AA after transplantation/AA fold change of differential KEGG level-3 metabolic pathways between AA and AA after transplantation. (**B**) Correlation networks showing the relationships between the differential microbial species and the differential microbial KEGG level-3 metabolic pathways. The node size and color (red circle, differential microbial species; gray circle, differential microbial metabolic pathways) are proportional to the mean relative abundance in the respective population. The lines’ width and color (red, positive; black, negative) are proportional to the correlation strength. Only significant correlations (*R* > 0.60 or < −0.60; *P* < 0.05) are displayed.

When comparing the identified KEGG pathways, 18 level-3 pathways, including 2 “Cellular processes” pathways, 2 “Environmental information processing” pathways, 7 “Genetic information processing” pathways, and 7 “Metabolism” pathways, were significantly enriched (*P* < 0.05; Fig. 5A) in the cecal microbiota of AA after transplantation, whereas 10 level-3 pathways, including 1 “Cellular processes” pathway and 9 “Metabolism” pathways, were significantly enriched (*P* < 0.05; Fig. 5A) in the cecal microbiota of AA. Moreover, in total, 48 KEGG level-3 “Metabolism” pathways related to amino metabolism, carbohydrate metabolism, lipid metabolism, metabolism of cofactors and vitamins, and energy metabolism pathways were observed in the cecal microbiota in AA and AA after transplantation (Fig. S2A through E). Among these “Metabolism” pathways, seven pathways, including “Galactose metabolism” (ko00052), “Ubiquinone and other terpenoid-quinone biosynthesis” (ko00130), “Riboflavin metabolism” (ko00740), and four other pathways, were significantly enriched in the cecal microbiota of AA after transplantation (*P* < 0.05; Fig. S2A through E), whereas seven pathways, including “Phenylalanine metabolism” (ko00360), “Arginine and proline metabolism” (ko00330), “Starch and sucrose metabolism” (ko00500), and four other pathways, were significantly enriched in the cecal microbiota of AA (*P* < 0.05; Fig. S2A through E).

### *Lactobacillus* species were significantly correlated with microbial metabolism pathways

To investigate the roles of the cecal microbiota in the skeletal muscle energy metabolism, the functions of amino acid metabolism, glucose metabolism, lipid metabolism, metabolism of cofactors and vitamins, and energy metabolism in the cecal microbiota were observed. A correlation network of different microbial species and different KEGG level-3 pathways related to the abovementioned metabolism was created to explore how cecal microbiota species affected these microbial functions. In total, 18 different microbial species showed significant associations with seven pathways (*P* < 0.05; *R* > 0.60; Fig. 5B). Among these positive relationships, five *Lactobacillus* species, including *L. amylovorus*, *L. ingluvie*i, *L. oris*, *L. plantarum*, and *L. vaginalis*, were more highly correlated (*P* < 0.05; *R* > 0.70; Fig. 5B) with the microbial “Porphyrin and chlorophyll metabolism” pathway.

### AA after transplantation and AA present significantly different metabolite profiles

**Fig 6:**
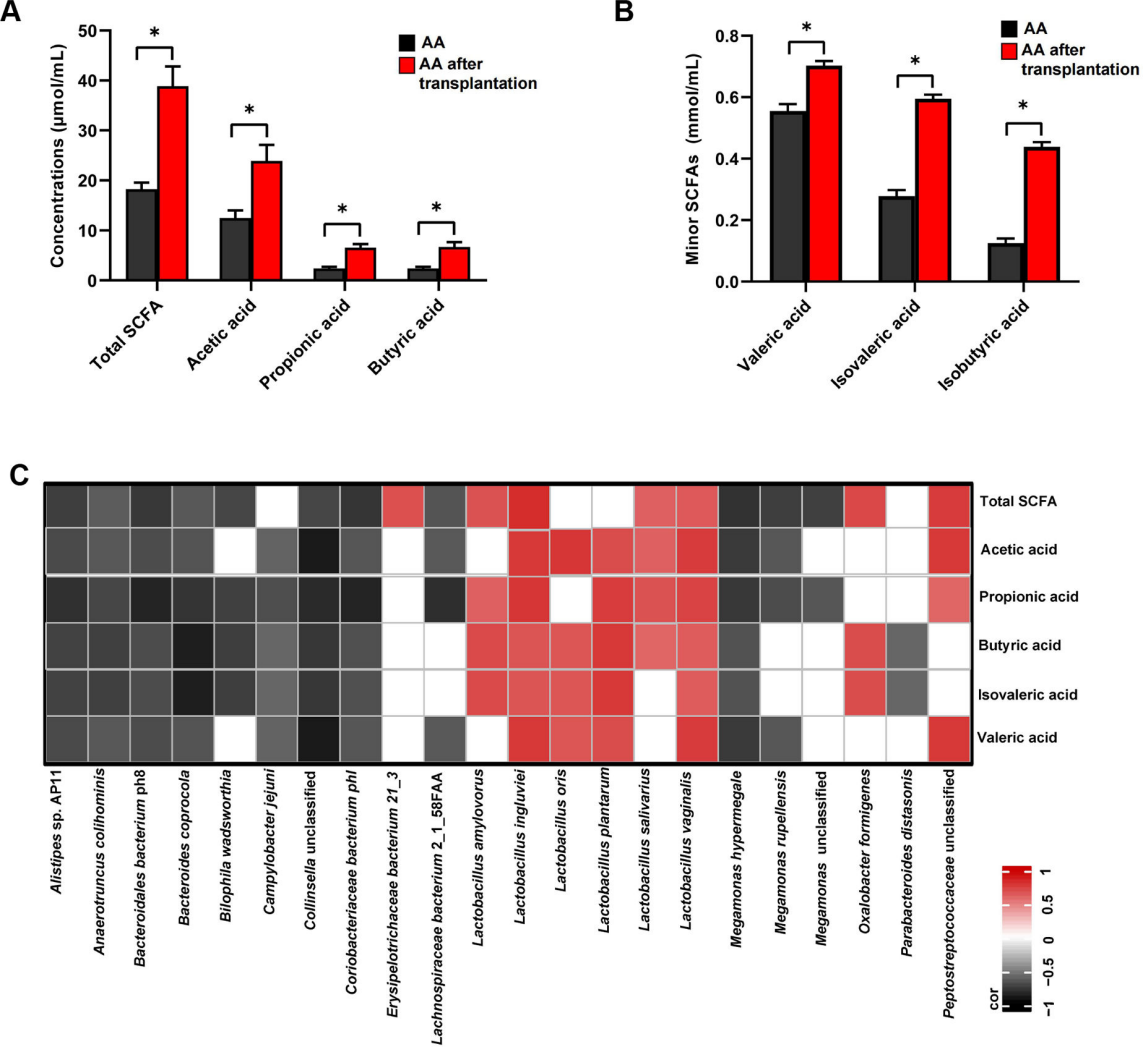
Concentrations of SCFAs in the cecum and their association with cecal microbial species. (**A**) The concentrations of the major SCFAs (total SCFAs, acetic acid, propionic acid, and butyric acid) and (**B**) minor SCFAs (valeric acid, isovaleric acid, and isobutyric acid) in the cecum of AA and AA after transplantation. *P < 0.05 between the indicated groups; the data are the means ± SEM (*n* = 6 chickens). (**C**) Interactions between the cecal differential SCFAs and cecal differential microbial species. Only strong correlations (*R* > 0.60 or *R* < −0.60, *P* < 0.05) are displayed in the heatmaps. The correlation scale ranged from −1 (black) to 1 (red).

To investigate the role of the gut microbiota in skeletal muscle energy metabolism in chickens, the concentrations of short-chain fatty acids (SCFAs) were quantified in the cecal contents. The concentrations of total SCFAs, acetic acid, propionic acid, butyric acid, valeric acid, isovaleric acid, and isobutyric acid in the cecum of AA after transplantation were significantly higher than those in AA (*P* < 0.05; Fig. 6A and B). In the correlation analysis between the significantly different cecal microbial species and cecal differential, SCFAs were significantly positively correlated with nine cecal microbial species (*P* < 0.05; *R* > 0.60), and these correlations mainly existed between six *Lactobacillus* species (*L. amylovorus, L. ingluviei, L. oris, L. plantarum, L. salivasrius,* and *L. vaginalis*) and the total SCFAs, acetic acid, propionic acid, butyric acid, valeric acid, and isovaleric acid (*P* < 0.05; 0.62 < *R* < 0.83; Fig. 6C).

**Fig 7:**
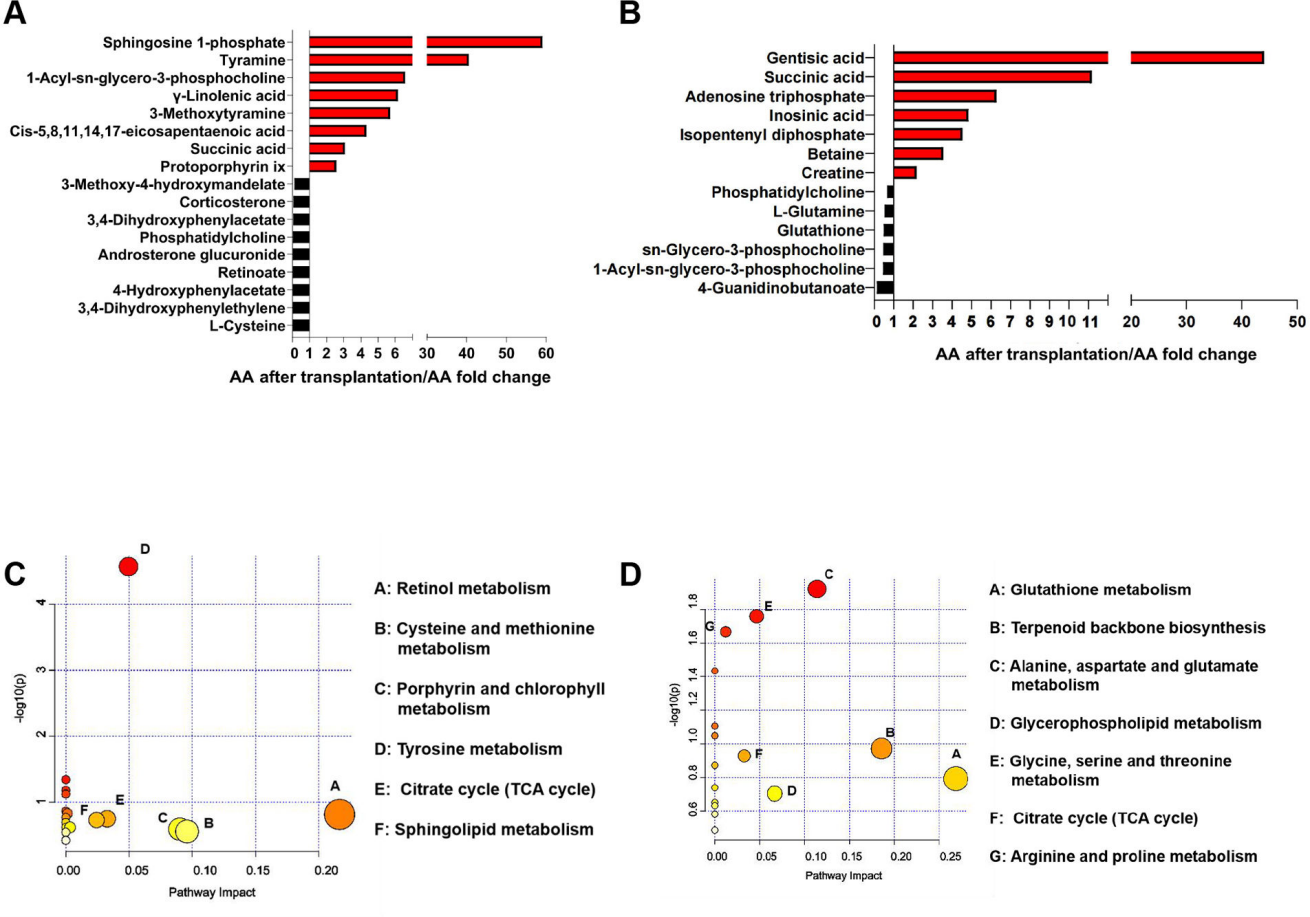
Serum and skeletal muscle metabolites of AA and AA after transplantation. (**A**) AA after transplantation/AA fold change of the significantly different serum metabolites between AA and AA after transplantation. (**B**) AA after transplantation/AA fold change of the significantly different metabolites in the pectoralis muscle between AA and AA after transplantation. (**C**) Pathway enrichment analysis of the differential serum metabolites between AA and AA after transplantation. (**D**) Pathway enrichment analysis of the differential metabolites in the pectoralis muscle between AA and AA after transplantation.

In the metabonomic analysis, a total of 1,130 compounds were identified in the serum of AA and AA after transplantation. The partial least squares discriminant analysis (PLS-DA) demonstrated a clear separation of the metabolites in the serum samples from AA and AA after transplantation (Fig. S3A), indicating significant differences in serum metabolites between AA and AA after transplantation. Based on the *t*-test, variable importance in the projection (VIP), and fold change filtering analyses of the relative concentrations, 309 different metabolites were identified between AA and AA after transplantation; of these, 139 metabolites were significantly higher in the serum of AA after transplantation, whereas the other 170 metabolites were significantly higher in the serum of AA than that of AA after transplantation (*P* < 0.05; VIP ≥ 1; fold change > 1.2 or < 0.83; Fig. S3B). The metabolic pathway analysis (MetPA) based on these significantly different serum metabolites revealed that 17 metabolites were enriched in 17 KEGG pathways (Fig. 7A and C), with the “Retinol metabolism,” “Cysteine and methionine metabolism,” and “Porphyrin and chlorophyll metabolism” pathways being significantly different pathways (*P* < 0.05; pathway impact > 0.05). The significantly different serum metabolites were subjected to a skeletal muscle fiber size (fiber diameter and density, FS) correlation analysis, and 66 FS-associated serum metabolites were detected (*P* < 0.05; 0.60 < *R* < −0.60; Table S3). The correlations between the significantly different cecal microbial species and serum FS-associated metabolites were assessed. There were 22 metabolites that were significantly correlated with 24 cecal microbiota (*P* < 0.05; *R* > 0.60), and positive correlations existed between 6 main *Lactobacillus* species (*L. amylovorus*, *L. ingluviei*, *L. oris*, *L. plantarum*, *L. saerimneri,* and *L. vaginalis*) and amino acids, peptides, analogs, fatty acids, organic acids, and porphyrins (*P* < 0.05; 0.60 < *R* < 0.89; Fig. S4A).

In the metabonomic analysis, a total of 1,126 compounds were identified in the pectoralis muscle of AA and AA after transplantation. The PLS-DA demonstrated a clear separation of the pectoralis muscle samples from AA and AA after transplantation (Fig. S3C), indicating significant differences in skeletal muscle metabolites between AA and AA after transplantation. Based on the *t* test, VIP, and fold change filtering analyses of the relative concentrations, 183 metabolites in the pectoralis muscle significantly differed between AA and AA after transplantation; of these, 119 metabolites were significantly higher in the pectoralis muscle of AA after transplantation, whereas the other 64 metabolites were significantly higher in the pectoralis muscle of AA (*P* < 0.05; VIP ≥ 1; fold change >1.2 or <0.83; Fig. S3D). The MetPA based on these significantly different metabolites revealed that 11 metabolites were enriched in 17 KEGG pathways (Fig. 7B and D), with the “Glutathione metabolism,” “Terpenoid backbone biosynthesis,” “Alanine, aspartate, and glutamate metabolism,” and “Citrate cycle (TCA cycle)” pathways being significantly different pathways (*P* < 0.05; pathway impact >0.05). The significantly different metabolites in the pectoralis muscle were subjected to a skeletal muscle fiber size (fiber diameter and density, FS) correlation analysis, and 39 FS-associated metabolites were detected (*P* < 0.05; 0.60 < *R* < −0.60; Table S4). The correlations between the different cecal microbial species and skeletal muscle FS-associated metabolites were assessed. Eighteen metabolites were significantly positively correlated with 23 cecal microbiota (*P* < 0.05; *R* > 0.60), and these correlations existed between seven main *Lactobacillus* species (*L. amylovorus, L. ingluviei, L. oris, L. plantarum, L. saerimneri, L. salivarius,* and *L. vaginalis*) and amino acids, peptides, analogs, organic acids, benzene and its derivatives (*P* < 0.05; 0.60 < *R* < 0.91; Fig. S4B).

To determine whether the FS-associated metabolites in serum could be related to those in the pectoralis muscle, the significantly different FS-associated metabolites in serum and the pectoralis muscle were compared. In total, three significantly different metabolites (3-methoxytyrosine, dodecanedioic acid, and succinic acid) were shared between the serum and pectoralis muscle of AA and AA after transplantation (Fig. S5A). Regarding the significantly different metabolite-enriched KEGG pathways, seven metabolic pathways related to “Amino acid metabolism,” “Metabolism of other amino acids,” and “Carbohydrate metabolism” were shared in both the serum and pectoralis muscle of AA and AA after transplantation (Fig. S5B).

### *Lactobacillus* regulates the fiber size and glucose metabolism in skeletal muscle in chickens

**Fig 8:**
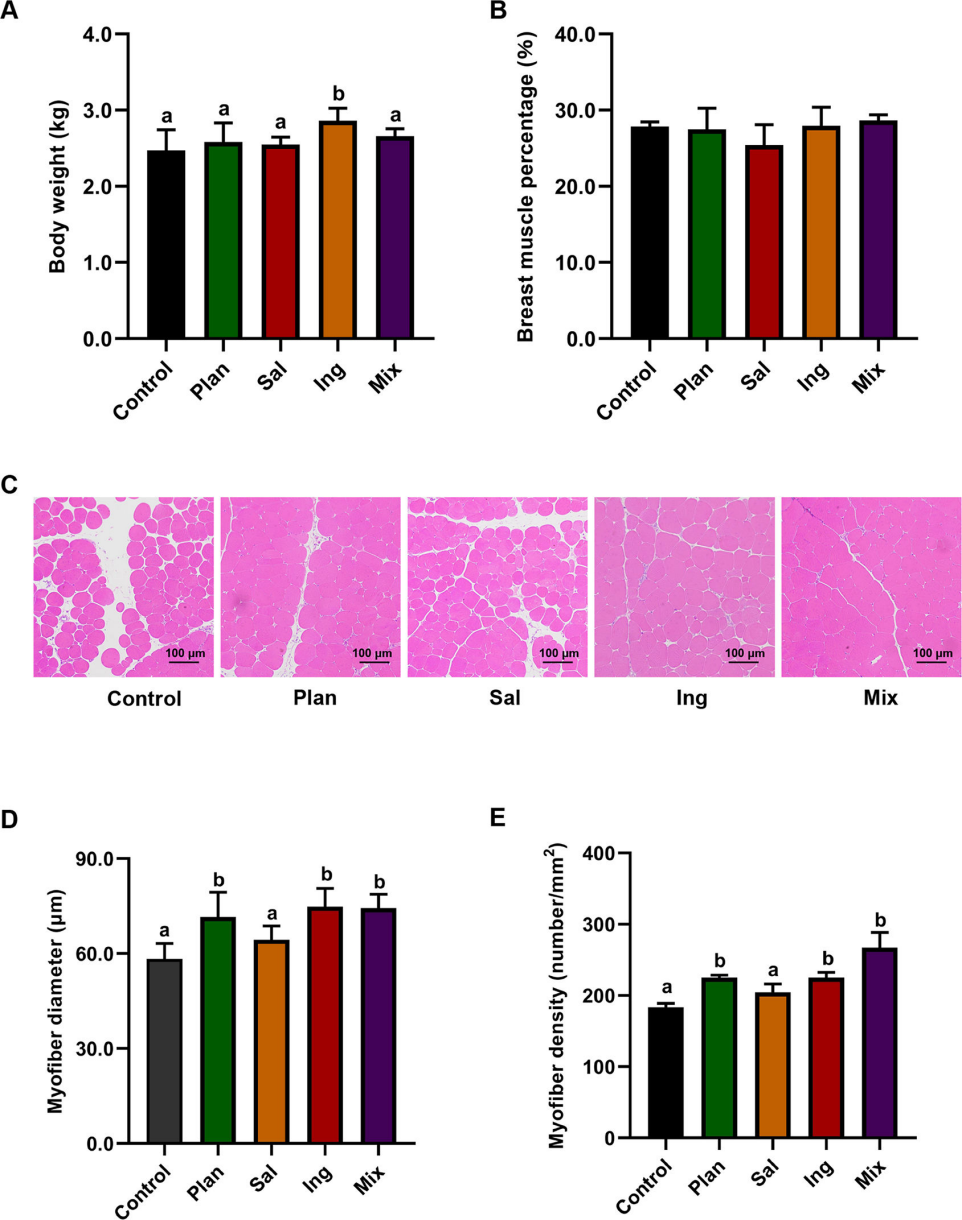
Body weight and skeletal muscle characteristics of chickens-fed *Lactobacillus*. (**A**) Body weight of Control, Plan, Sal, Ing, and Mix. (**B**) The breast muscle percentage of Control, Plan, Sal, Ing, and Mix. (**C**) HE staining (original magnification, ×100) showing the fiber size in the pectoralis muscle of Control, Plan, Sal, Ing, and Mix. (**D**) The pectoralis muscle fiber diameters and (**E**) densities of Control, Plan, Sal, Ing, and Mix. The chickens in group Control received sterilized PBS, and the chickens in groups Plan, Sal, Ing, and Mix received 10^8^ CFU/mL *L*. *plantarum*, *L. salivariu*sm, *L. ingluviei*, or a mixed bacterial suspension (1:1:1 mixed) as described below. In the figure, different letters indicate significant differences (*P* < 0.05); the letters indicate no significant difference. The data are the means ± SEM (*n* = 12 chickens).

To assess the potential function of *Lactobacillus* species on glucose metabolism in the skeletal muscle of chickens, we designed a feeding experiment. The results showed that the body weights were significantly increased in the Ing and Mix groups (*P* < 0.05; Fig. 8A) compared to Control and Plan. No differences in the breast muscle percentage ratio were observed among the Control, Plan, Sal, Ing, and Mix (*P* > 0.05; Fig. 8B) compared to control. The fiber size of the pectoralis muscle was observed using hematoxylin-eosin staining (Fig. 8C). Compared with the control, the fiber diameter was increased in the Plan, Ing, and Mix groups (*P* < 0.05; Fig. 8D). Moreover, the fiber density of the pectoralis muscle was significantly increased in Plan, Ing, and Mix groups compared with that in the control (*P* < 0.05; Fig. 8E).

**Fig 9:**
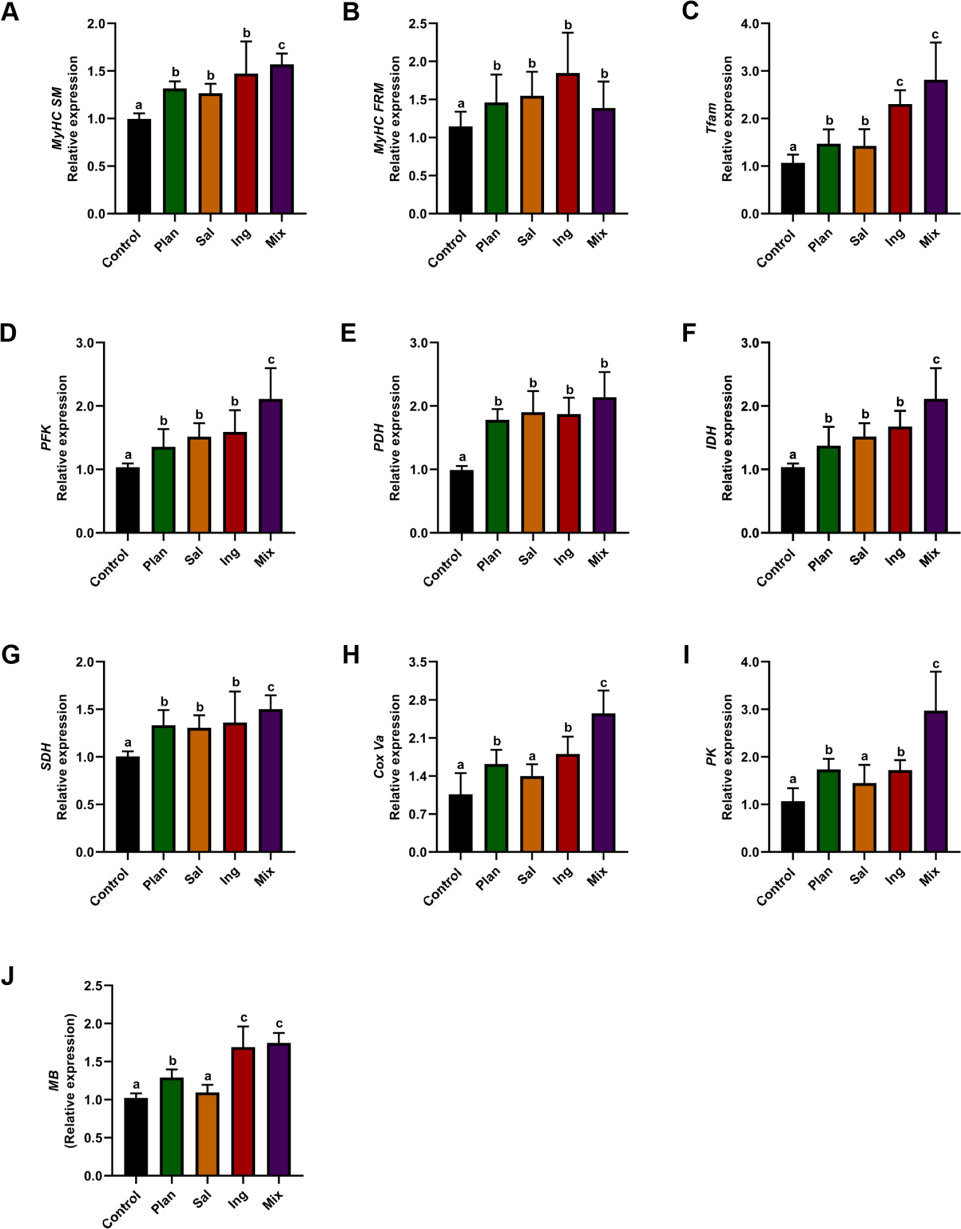
Gene expression in skeletal muscle of chickens-fed *Lactobacillus*. The expression of genes encoding myosin heavy chain (MyHC) isoforms. (**A**) *MyHC SM*, (**B**) *MyHC FRM*, (**C**) Myoglobin (*MB*), (**D**) *Tfam,* (**E**) *CoxVa,* (**F**) PFK, (**G**) PK, (**H**) PDH, (**I**) IDH, and (**J**) SDH genes in the pectoralis muscle of Control, Plan, Sal, Ing, and Mix (*n* = 6). In the figure, different letters indicate significant differences (*P* < 0.05); the letters indicate no significant difference. The data are the means ± SEM (*n* = 12 chickens).

Subsequently, our data showed that the level of glucose metabolism was enhanced in skeletal muscle after gavage with *L. plantarum*, *L. ingluviei*, *L. salivarius,* and their mixture. The expression levels of the *MyHC SM*, *MyHC FRM*, *Tfam*, *PFK*, *PDH*, *IDH*, and *SDH* genes were significantly increased in the pectoralis muscle in each test group compared to those in the control (*P* < 0.05; Fig. 9A through G). The expression levels of the *CoxVa*, *PK*, and *MB* genes were significantly increased in the pectoralis muscle of the Plan, Ing, and Mix groups compared to those in the control (*P* < 0.05; Fig. 9H through J).

**Fig 10:**
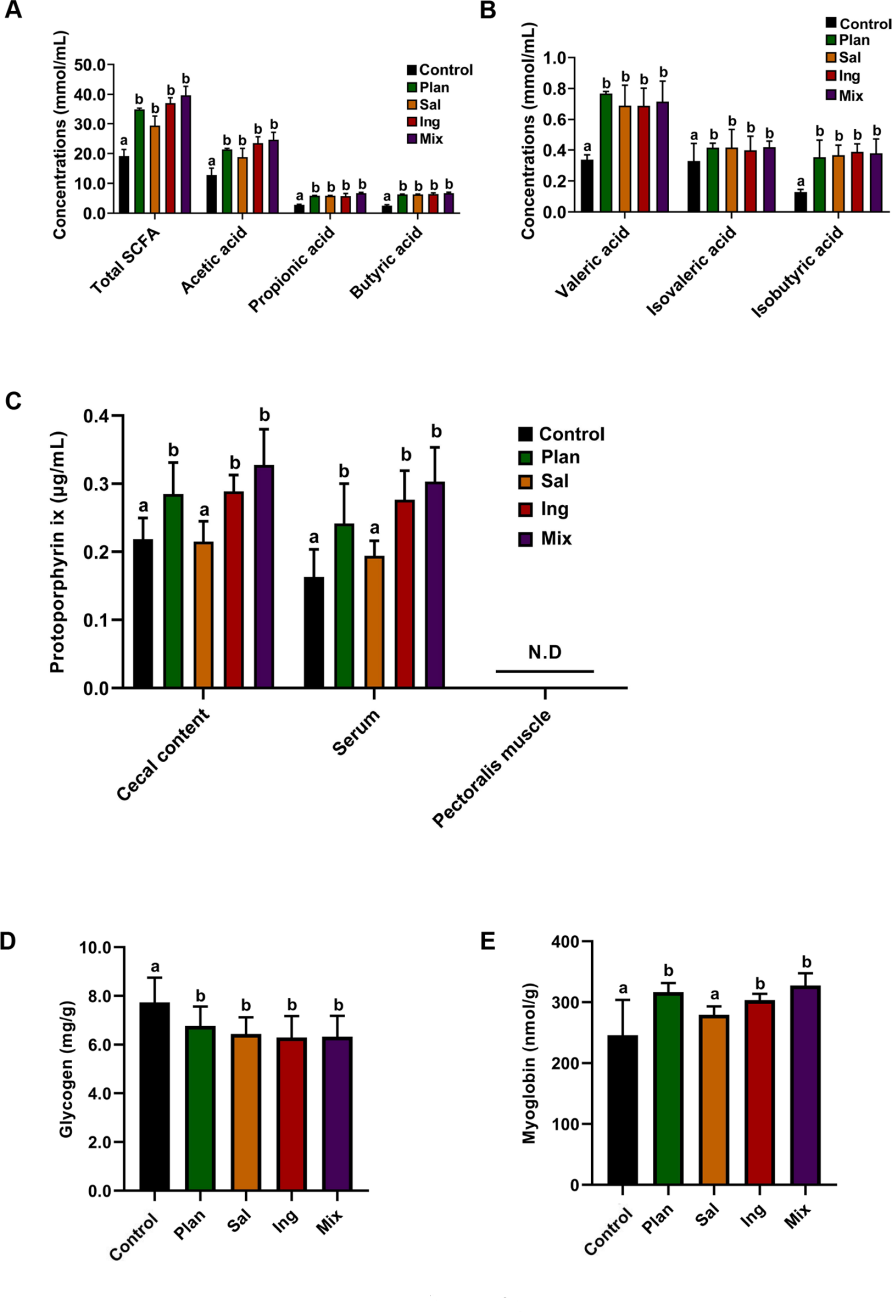
Concentrations of key metabolites in the cecal contents, serum, and pectoralis muscle of chickens-fed *Lactobacillus*. (**A**) Concentrations of the major SCFAs (total SCFAs, acetic acid, propionic acid, and butyric acid) in the cecum of Control, Plan, Sal, Ing, and Mix. (**B**) Concentrations of minor SCFAs (valeric acid, isovaleric acid, and isobutyric acid) in the cecum of Control, Plan, Sal, Ing, and Mix. Concentrations of (**C**) protoporphyrin IX, (**D**) glycogen, and (**E**) myoglobin in the pectoralis muscle of the Control, Plan, Sal, Ing, and Mix. In the figure, different letters indicate significant differences (*P* < 0.05); the letters indicate no significant difference. N.D, not detected. The data are the means ± SEM (*n* = 12 chickens).

Finally, key metabolites related to skeletal muscle energy metabolites were observed. The concentrations of total SCFAs, acetic acid, propionic acid, butyric acid, valeric acid, isovaleric acid, and isobutyric acid in the cecal content of each test group were significantly increased compared to those in the control (*P* < 0.05; Fig. 10A and B). The concentrations of myoglobin and protoporphyrin IX in the pectoralis muscle of Plan, Ing, and Mix groups were significantly higher than those in the Control (*P* < 0.05; Fig. 10C and E). The concentration of glycogen was significantly decreased in the pectoralis muscle in each test group compared to that in Control (*P* < 0.01; Fig. 10D).

## DISCUSSION

This study shows that the gut microbiota contributes to the fiber size and glucose metabolism in skeletal muscle of chickens. *L. plantarum, L. ingluviei,* and *L. salivarius* play vital roles in the production of microbial protoporphyrin IX and SCFAs. Protoporphyrin IX enters the blood and then synthesizes myoglobin, which uses oxygen to convert stored energy. SCFAs provide energy to skeletal muscle through intestinal mucosal absorption, and microbial SCFAs improve muscle energy metabolism (15, 17).

In this study, the cecal microbiota-recipient AA chickens showed similar skeletal muscle characteristics, including increased fiber diameter and expression levels of genes related to oxidative fiber type, mitochondrial function, and glucose metabolism, which tended to be consistent with those of cecal microbiota donor JY. These results are similar to previous studies involving mammals, which showed that fecal microbiota transplantation from pigs to mice or from mice to mice could alter the fiber diameter and density in gut microbiota recipients (3, 18). According to the KEGG functional analysis of the cecal microbiota, the “Porphyrin and chlorophyll metabolism” pathway was enriched in the cecal microbiota of AA after transplantation compared with that of AA, and *L. plantarum, L. ingluviei,* and *L. salivarius* with a higher abundance in AA after transplantation showed a higher positive correlation with this pathway. This result suggests that these *Lactobacillus* species may have the potential to produce more protoporphyrin IX. Coincidentally, the metabonomics analysis revealed that the abundance of protoporphyrin IX in the serum of AA after transplantation was significantly higher than that of AA. Protoporphyrin IX, as a key precursor in the heme biosynthetic pathway, along with its Fe^2+^ complex (heme) plays a crucial role as important cofactors of myoglobin for mitochondrial function and oxidative stress response, which are crucial for muscle metabolism and adaptation (19). In this study, further investigations found that *MB* gene expression and the myoglobin concentration in the pectoralis muscle of AA after transplantation were significantly higher than those of AA, suggesting that cecal microbial protoporphyrin IX participates in myoglobin biosynthesis in the pectoralis muscle of chicken hosts and that the production of this substance in the pectoralis muscle of AA after transplantation was greater than that of AA. A higher concentration of myoglobin may reflect an increased oxidative capacity of skeletal muscle, potentially enhanced glucose metabolism in the pectoralis muscle of AA after transplantation (20). Intriguingly, 5-aminolevulinic acid, a precursor to protoporphyrin IX, has been shown to enhance glucose uptake in skeletal muscle, providing evidence that protoporphyrin IX could influence glucose transporter expression and activity and glucose metabolism (21), would provide an elevated supply of energy for muscle mass enhancement.

In the cecum, microbiota utilize nondigestible carbohydrates to produce SCFAs, which have important effects on the energy metabolism of the host (22, 23). In this study, cecal SCFAs, including the concentrations of total SCFA, acetic acid, propionic acid, butyric acid, valeric acid, isovaleric acid, and isobutyric acid, in the cecum of AA after transplantation were significantly higher than those of AA. A previous study reported that SCFAs produced by gut microbiota can be absorbed by the host and then converted into glucose (24). Moreover, a previous study reported that after entering skeletal muscle cells, gut microbial metabolite SCFAs act as ligands for the fatty acid receptors FFAR-2 and FFAR-3 to promote glucose intake and metabolism and increase insulin sensitivity (22). Furthermore, the major target of SCFAs in skeletal muscle is mitochondria. SCFAs can upregulate the NAD-dependent protein deacetylase sirtuin-1, which is the regulatory factor for mitochondrial biogenesis (25). It has been suggested that compared with AA, the cecal microbiota of AA after transplantation provides more glucose to the host by producing SCFAs and enhancing the metabolism in their skeletal muscle. In this study, most of the microbiota species, which showed significantly higher relative abundances in the cecum of AA after transplantation, belonged to the *Lactobacillus* genus. This genus is among the most important microbial genera in the gut of chickens and is able to produce SCFAs (26, 27). The correlation analysis revealed that *L. plantarum*, *L. ingluviei*, and *L. salivarius* were highly positively correlated with cecal SCFAs. Moreover, these microbes can promote SCFA production in the gut as verified by the microbe-feeding experiments. These results suggest the essential roles of these microbes in SCFA production and imply their potential roles in glucose metabolism in skeletal muscle (28).

In this study, succinic acid, another important metabolite, was a common differential metabolite in serum and skeletal muscle. Succinic acid is an important tricarboxylic acid (TCA) cycle intermediate and a fuel compound for mitochondrial oxidative phosphorylation (29). In this study, skeletal muscle with increased glucose metabolism (the pectoralis muscle of AA after transplantation) had a higher succinic acid abundance. This result is consistent with a previous study showing that succinic acid enhanced skeletal muscle energy metabolism by increasing aerobic enzyme activity, oxygen consumption, and mitochondrial biogenesis in skeletal muscle (29). Furthermore, succinic acid increased myosin heavy chain I expression and induced skeletal muscle fiber-type remodeling by SUNCR1 signaling and its downstream calcium/NFAT signaling pathway (30). In addition, succinic acid promotes skeletal muscle protein synthesis via the Erk1/2 signaling pathway, and an increase in protein synthesis can cause hypertrophy of muscle fibers (31, 32). In this study, AA after transplantation had a larger pectoralis muscle fiber diameter than AA. The higher abundance of succinic acid in the skeletal muscle of AA after transplantation may contribute to the larger fiber diameter. Succinic acid can be produced by the gut microbiota, rendering it an especially important metabolite among gut microbiome-host metabolic interactions (29). *Lactobacillus* species were reported to be able to produce succinic acid in previous studies (33, 34). In this study, the abundance of seven *Lactobacillus* species was higher in the cecum of AA after transplantation than that in AA, which may contribute to the higher abundance of succinic acid in the serum and skeletal muscle of AA after transplantation. However, there were no correlations between the cecal *Lactobacillus* species and microbial metabolic pathways related to microbial succinic acid production. Therefore, in this study, *Lactobacillus* species were unlikely to produce succinic acid in the cecum. Therefore, other speculation suggests that the cause of the increased succinic acid abundance in the pectoralis muscle and serum was the increased myoglobin concentration in skeletal muscle. Increased myoglobin facilitates more oxygen entry into muscle fibers, enhances their oxidative capacities, and intensifies energy metabolism through the TCA cycle (35), which may increase the levels of its intermediate, succinic acid, in skeletal muscle. In this study, a higher expression of the *SDH* gene was observed in the pectoralis muscle of AA after transplantation compared to AA. This result suggests that the metabolism of succinic acid to fumaric acid is also enhanced in the pectoralis muscle and that the process is stronger in AA after transplantation, leading to the release of more energy in the pectoralis muscle (36). Moreover, succinic acid in the pectoralis muscle could enter the blood circulation, causing it to increase in the blood (37).

### Conclusion

In conclusion, *L. plantarum, L. ingluviei*, and *L. salivarius* could regulate glucose metabolism in skeletal muscle through their metabolites, protoporphyrin IX and SCFAs (together with host metabolites), myoglobin, and succinic acid and by inducing differential sizes of skeletal muscle in chickens. This study is vital for the development of strategies for regulating the metabolism of skeletal muscle in chickens by manipulating the gut microbiota to improve chicken quality in the future.

## MATERIALS AND METHODS

### Animals

The cecal microbiota donors were six male Chinese indigenous Jingyuan chickens and six male Arbor Acres chickens aged 180 and 42 days, respectively. These chickens had similar market body weights and were obtained from a farm located in Jingning County of Gansu Province, China. The diet formula was designed based on the Chinese *Feeding Standard of Chicken (NY/T 33-2004)*. The compositions of the diets for JY and AA are provided in Tables S5 and S6, respectively. Feeds were given at 6:00 am and 1:00 pm, with both JY and AA having free access to feed and water in a separated 200 m^2^ area.

### Cecal microbiota transplantation

The suspension of cecal content was prepared as described by Hu et al. (38). In brief, the cecal contents were homogenized in sterilized saline and then passed through four layers of sterilized medical gauze and a 0.22-mm stainless cell strainer to eliminate large and small particles. Subsequently, the live microbes in the suspension were counted by using optical microscopy (Olympus Corporation, Japan). Then, sterile glycerol was added to the suspension at a final concentration of 10% (vol/vol), and the suspension was stored at −80°C. In total, 48 21-day-old AA chickens with similar body weights were randomly divided into two groups, the AA and AA after transplantation, with six replicate cages of 4 chickens per replicate cage. The chickens in the AA group received sterilized PBS, and the chickens in the AA after transplantation group received a 10^8^ CFU/mL suspension of cecal content by gavage at a volume of 1.0 mL from the age of 22–42 days. For sampling, one bird from each replicate with a similar body weight was selected after 12 h of overnight fasting.

### *Lactobacillus* feeding experiments

To deplete the gut microbiota, the chickens were administered 1.0 mg/mL ampicillin, 0.5 mg/mL vancomycin, and 1.0 mg/mL metronidazole in the drinking water for 10 days (from the age of 12–21 days). In total, 120 22-day-old AA chickens with similar body weights were randomly divided into 5 groups with 6 replicate cages per group, each containing 4 chickens. The chickens in the Control group received sterilized PBS, and the chickens in the Plan, Sal, Ing, and Mix groups received 10^8^ CFU/mL *L*. *plantarum*, *L. salivarius*, *L. ingluviei,* or a mixed bacterial suspension (1:1:1 mixed), respectively, by gavage at 1.0 mL/day from the age of 22–42 days. One bird per replicate with a similar body weight was selected after 12 h of overnight fasting.

### Sample collection

Blood was drawn from the wing vein, placed into tubes, and centrifuged at 3,000 × *g* for 15 min to separate the serum. The chickens were euthanized via carotid artery exsanguination, and the cecal contents and skeletal muscle were collected and transferred into sterile centrifuge tubes. The samples were immediately frozen in liquid nitrogen and then stored at −80°C until further analysis. For the tissue sectioning, skeletal muscle was collected from the pectoralis major and then fixed with 4% paraformaldehyde and 2.5% glutaraldehyde.

### Histological assessment of skeletal muscle

Muscle sections were stained with hematoxylin-eosin. Five fields per sample were observed and photographed under an Olympus BX53 microscope (Olympus Corporation, Japan). For the TEM observation, fixed pectoralis muscle samples were treated with 1% osmium tetroxide (precooling treatment at 4°C), dehydrated in ethanol, and embedded in epoxy resin. Finally, 60–80 nm sections were cut by an EMUC7 ultramicrotome (Leica, Germany) and stained with uranyl acetate and lead citrate. Five fields of view per sample were observed under a Tecnai G^2^20-TWIN transmission electron microscope (FEI company, USA) at 5,000× magnification. ImageJ software (National Institutes of Health, USA) was used to measure the fiber diameter and field size and count the number of fibers and mitochondria.

### Gene expression analysis by real-time quantitative PCR

The total RNA was extracted from the pectoralis muscle using TRIzol Reagent (Life Technologies, USA). The purity and integrity of RNA were assessed by a NanoDrop 2000 (Thermo Scientific) and agarose gel electrophoresis. Then, the RNA was reverse transcribed using a cDNA Synthesis Kit (ABclonal, RK20429). Primers were designed using Primer 5.0 software (Primers Co. Ltd., Canada), and the primer sequences are shown in Table S7. The primers were synthesized by Sangon Biotech Co., Ltd. (Shanghai, China). The efficiencies of PCR were acquired according to the formula 10^(−1/slope)^, and the efficiencies of all primers used in this study were between 96% and 105% (39). RT-PCR was conducted by using a SYBR Green Mix (RK21206, ABclonal, China) in conjunction with an ABI QuanStudio TM6 flex real-time fluorescent quantitative PCR system (Life Technologies, USA). The thermocycler conditions were as follows: 95°C for 5min, 40 cycles of 95°C for 15s, 60°C for 30s, and 72°C for 30s. In addition, melting curves were collected from 60°C to 90°C. The geometric mean of hydroxymethylbilane synthase (HMBS) and β-actin (geNorm *M* < 0.50, *V*2/3 < 0.15 threshold) was used as the internal control to normalize the amount of starting RNA used in the RT-PCR, and the relative expression levels were quantified using the 2^−ΔΔCt^ method (40).

### Measurements of glycogen, myoglobin, and protoporphyrin IX

The glycogen concentration in the pectoralis muscle samples was measured using a Glycogen Assay Kit (Nanjing Jiancheng Bioengineering Institute, China) following the manufacturer’s instructions.

The myoglobin concentration in the pectoralis muscle was determined by spectrophotometry as described by Ma et al. (41). In brief, the pectoralis muscle sample was homogenized in buffer (3.0 mmol/L MgCl_2_, 5.0 mmol/L EDTA, 75 mmol/L Tris, pH = 7.2) and centrifuged at 4°C and 10,000 × *g* for 10 min. The supernatant was detected by a UV-2100 spectrophotometer (UNIC, China). The myoglobin concentration was calculated as follows:


$$C_{Mb}=\left(A_{576}\times V_{s}\right)/(12.8\times Ws)$$

where *A*_576_ is the absorbance value of the supernatants at 576 nm, *V*_*s*_ is the supernatant volume, 12.8 is the millimolar extinction coefficient at 576 nm, and Ws is the weight of the pectoralis muscle sample.

The protoporphyrin IX concentrations in the cecal content, serum, and pectoralis muscle samples were determined by high-performance liquid chromatography (HPLC) as described by Wakamatsu et al. (42). In brief, the mobile phase of HPLC was methanol-ammonium acetate (86:14 [vol/vol], pH 5.16) at a flow rate of 0.6 mL/min at 35℃. A Hypersil gold C18 column (250 × 4.6 mm, 5 µm; Thermo Fisher Scientific, USA) was used for the separation of protoporphyrin IX. Then, 20 µL of each sample was injected. The detection of protoporphyrin IX was carried out at excitation and emission wavelengths of 590 and 630 nm, respectively.

### Detection of SCFAs by gas chromatography

The concentrations of SCFAs were determined by gas chromatography as described by Chang et al. (43). In brief, 2.0 g of cecal content was diluted in 5.0 mL of ultrapure water, vortexed for 3–5 min, and centrifuged at 5,000 × *g* for 10 min. One milliliter of supernatant from the samples was combined with 0.25 mL of 25% (wt/vol) metaphosphoric acid and centrifuged at 10,000 × *g* for 10 min. The mixture was then injected into a Chrompack CP-Wax 52 fused silica column (30 m × 0.53 mm × 1.00 µm) of a gas chromatograph equipped with a Model 2010 flame ionization detector (Shimazu, Japan).

### DNA extraction, metagenome sequencing, and metagenomics data processing

DNA was extracted from the cecal contents of each individual chicken using a commercially available QIAamp Fast DNA Stool Mini Kit (Qiagen, Germany), following the manufacturer’s instructions. The quality and quantity of DNA were measured using a NanoDrop ND-1000 Spectrophotometer (Thermo Fisher Scientific, Massachusetts, USA). Genomic DNA (1.0 µg) was randomly fragmented by Covaris. Fragmented DNA with an average size of 300–400 bp was selected by magnetic beads. The selected DNA fragments were subjected to end repair, 3′’ adenylation, adapter ligation, and PCR amplification, and the products were purified by magnetic beads. The double-stranded PCR products were heat denatured and circularized by the splint oligo sequence. The final format of the library was single-stranded circular DNA, which was checked by QC. Qualified library sequencing was performed on a BGIseq-500 platform (BGI, China). SOAPnuke v.1.5.2 (44) was used for the raw data trimming. The trimmed reads were mapped to the host genome using SOAP2 software (45) to identify and remove host-originated reads. High-quality reads were *de novo* assembled using IDBA-UD software (46). Assembled contigs with lengths less than 300 bp were discarded from further analysis. Genes were predicted over contigs by using MetaGeneMarker v.2.10 (47). Redundant genes were removed using CD-HIT with an identity cutoff of 95% (48).

### Taxonomic and functional annotation of cecal metagenomes

To generate the taxonomic information, the protein sequences of genes were aligned against the NR database using DIAMOND with an *E* value cutoff of 1e^−5^ (49). Based on the MEGAN LCA algorithm (50), taxonomic annotation was assigned. The taxonomic profiling was conducted at the kingdom, phylum, genus, and species levels, and the relative abundances were calculated. The α-diversity was quantified by the Shannon index using the relative abundance profiles at the microbial species level with R software. A PCA based on the Bray-Curtis distance at the microbial species level was performed, and the results were plotted with the R software package “ade4.” Microbial taxa with a relative abundance >0.01% in at least 50% of the chickens in each group were used for the downstream analysis.

To obtain functional information, the protein sequences were aligned against the KEGG database (89.1) by DIAMOND with an *E* value cutoff of 1e^−5^ (49). The PCA based on the Bray-Curtis distance of the KEGG pathways was plotted with the R software package “ade4.” KEGG pathways with a relative abundance >0.01% in at least 50% of the chickens in each group were used for the downstream analysis.

### Untargeted metabolomics analysis of serum and skeletal muscle

The serum metabolome and skeletal muscle metabolome were analyzed using a 2D UPLC chromatograph (Waters, USA) combined with a Q-Exactive HF mass spectrometer (Thermo Fisher Scientific, USA). Compound Discoverer 3.0 (Thermo Fisher Scientific, USA) was used for the peak extraction, peak alignment, and compound identification. The metabolomics analysis, which included data preprocessing, statistical analysis, metabolite classification, and functional annotation, was performed by the “metaX” package in R software (51). Metabolite peaks present in >50% of the samples with a relative standard deviation <30% or a similarity value >200 were retained (52). The identified peaks were used for the downstream analysis. The identification of metabolites was based on the combined results of the HMDB and KEGG databases. Based on the KEGG database, enrichment analysis and pathway analysis were performed by an online platform, MetaboAnalyst 5.0 (https://www. Metaboanalyst.ca).

### Correlation analysis

To explore the potential functional roles of gut microbiota, a correlation analysis between the significantly different microbial species and KEGG pathways was performed by using a Spearman’s rank correlation analysis. To identify the microbiota-associated metabolites, a correlation analysis was performed between the significantly different microbial species and cecal, serum, and skeletal muscle metabolites by using a Spearman’s rank correlation analysis using the “Hmisc” package (v.4.4-0) in R software. The correlations were considered significant when *P* values <0.05 and coefficients (*R*) >0.60 or <−0.60. The correlation network was visualized by Cytoscape (v.3.7.2), and correlation heatmaps were generated with the “ComplexHeatmap” package (v.2.4.3) in R software (v.4.0.2) (53).

### Statistical analysis

The “stats” package in R software (v.4.0.2) was used for the statistical analysis. The fiber diameter and density parameters of skeletal muscle, gene expression, and concentrations of SCFAs, glycogen, and myoglobin were compared between AA and JY or AA and AA after transplantation by using two-tailed Student’s *t* tests. The cecal microbiota was compared between AA and AA after transplantation at the kingdom, phylum, genus, and species levels by using a Wilcoxon rank-sum test, and *P* values <0.05 were considered statistically significant. The abundances of microbial KEGG metabolic pathways were compared between AA and AA after transplantation by using a Wilcoxon rank-sum test, and *P* values <0.05 were considered statistically significant. MetaboAnalyst 5.0 (https://www.metaboanalyst.ca/) was used to perform the pathway analysis of the metabolome data. A *t* test, variable importance in projection, and fold change measurements were used to compare the serum and skeletal muscle metabolites between AA and AA after transplantation, and a *P* value of <0.05, VIP of ≥1, and fold change of >1.2 or <0.83 were indicative of significantly different metabolites.

## Data Availability

The cecal metagenome sequences were deposited into the NCBI Sequence Read Archive (SRA) under the accession number PRJNA731973.

## SUPPLEMENTAL MATERIAL

Supplemental figures msystems.00214-24-s0001.pdf Figures S1 to S5.

Supplemental Material msystems.00214-24-s0002.docx Legends for supplemental figures and tables.

Table S1 msystems.00214-24-s0003.doc Summary of the sequence data generated from the cecum samples of AA and AA after transplantation.

Table S2 msystems.00214-24-s0004.xls Composition of cecal microbial metabolic pathways based on level 1-3 KEGG functions.

Table S3 msystems.00214-24-s0005.xls FS-associated differential metabolites in serum.

Table S4 msystems.00214-24-s0006.xls FS-associated differential metabolites in pectoralis muscle.

Table S5 msystems.00214-24-s0007.doc The composition of diets for Jingyuan chickens.

Table S6 msystems.00214-24-s0008.doc The composition of the diets of Arbor Acres chickens.

Table S7 msystems.00214-24-s0009.doc Primer sequences.

ASM does not own the copyrights to Supplemental Material that may be linked to, or accessed through, an article. The authors have granted ASM a non-exclusive, world-wide license to publish the Supplemental Material files. Please contact the corresponding author directly for reuse.
