# Supplementary material for: Lactobacillus-derived protoporphyrin IX and SCFAs regulate the fiber size via glucose metabolism in the skeletal muscle of chickens
Source: mSystems. 2024 May 23;9(6):e00214-24. doi: 10.1128/msystems.00214-24 (PMC11237663; doi:10.1128/msystems.00214-24)
Supplement: Supplemental figures — Figures S1 to S5. [file msystems.00214-24-s0001.pdf]

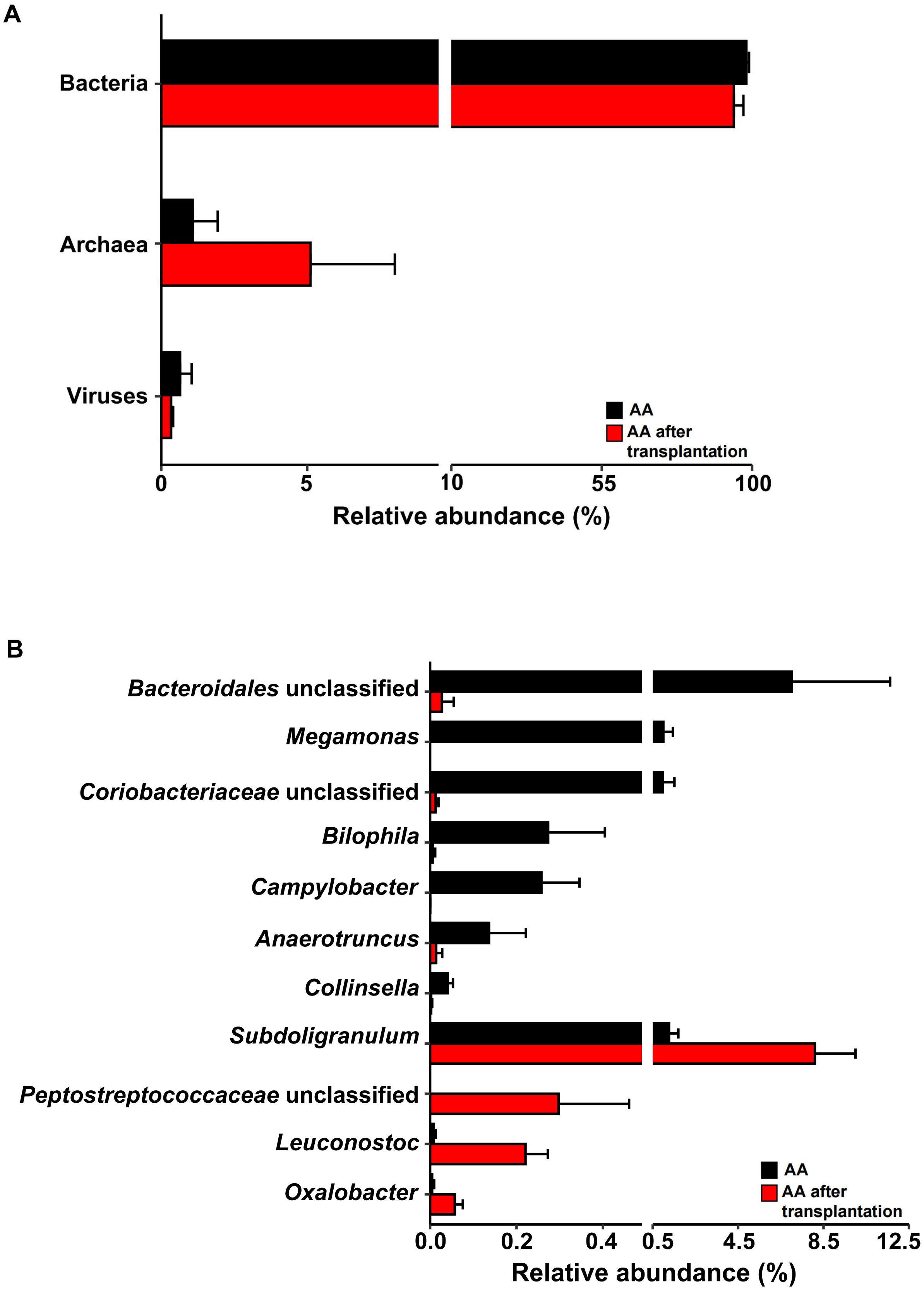

A
Bacteria
Archaea
Viruses
AA
AA after transplantation
0
5
10
55
100
Relative abundance (%)
B
Bacteroidales unclassified
Megamonas
Coriobacteriaceae unclassified
Bilophila
Campylobacter
Anaerotruncus
Collinsella
Subdoligranulum
Peptostreptococcaceae unclassified
Leuconostoc
Oxalobacter
AA
AA after transplantation
0.0
0.2
0.4
0.5
4.5
8.5
12.5
Relative abundance (%)

Figure S1

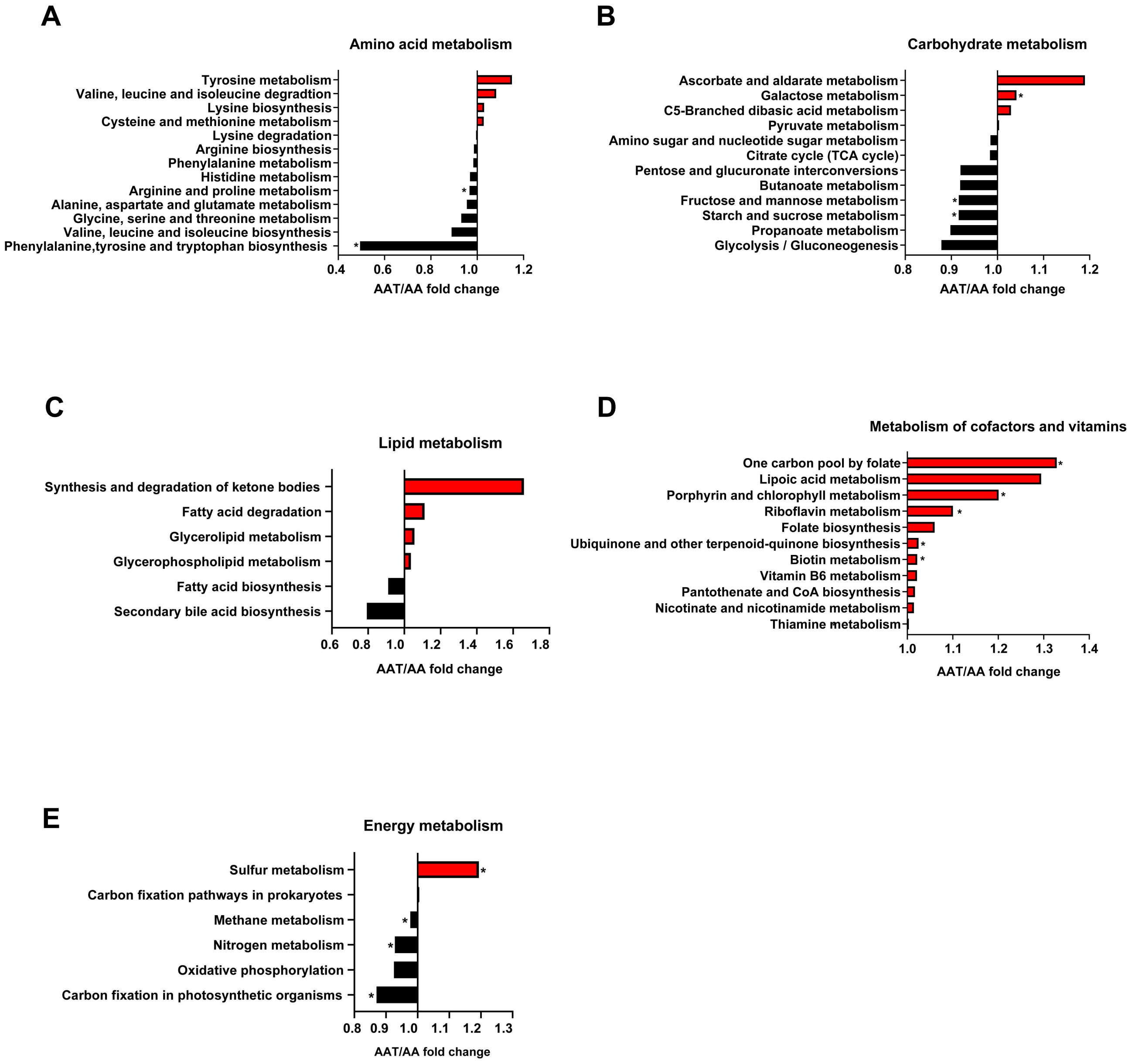
A
Amino acid metabolism
Tyrosine metabolism
Valine, leucine and isoleucine degradtion
Lysine biosynthesis
Cysteine and methionine metabolism
Lysine degradation
Arginine biosynthesis
Phenylalanine metabolism
Histidine metabolism
Arginine and proline metabolism
Alanine, aspartate and glutamate metabolism
Glycine, serine and threonine metabolism
Valine, leucine and isoleucine biosynthesis
Phenylalanine,tyrosine and tryptophan biosynthesis
0.4 0.6 0.8 1.0 1.2
AAT/AA fold change
B
Carbohydrate metabolism
Ascorbate and aldarate metabolism
Galactose metabolism
C5-Branched dibasic acid metabolism
Pyruvate metabolism
Amino sugar and nucleotide sugar metabolism
Citrate cycle (TCA cycle)
Pentose and glucuronate interconversions
Butanoate metabolism
Fructose and mannose metabolism
Starch and sucrose metabolism
Propanoate metabolism
Glycolysis / Gluconeogenesis
0.8 0.9 1.0 1.1 1.2
AAT/AA fold change
C
Lipid metabolism
Synthesis and degradation of ketone bodies
Fatty acid degradation
Glycerolipid metabolism
Glycerophospholipid metabolism
Fatty acid biosynthesis
Secondary bile acid biosynthesis
0.6 0.8 1.0 1.2 1.4 1.6 1.8
AAT/AA fold change
D
Metabolism of cofactors and vitamins
One carbon pool by folate
Lipoic acid metabolism
Porphyrin and chlorophyll metabolism
Riboflavin metabolism
Folate biosynthesis
Ubiquinone and other terpenoid-quinone biosynthesis
Biotin metabolism
Vitamin B6 metabolism
Pantothenate and CoA biosynthesis
Nicotinate and nicotinamide metabolism
Thiamine metabolism
1.0 1.1 1.2 1.3 1.4
AAT/AA fold change
E
Energy metabolism
Sulfur metabolism
Carbon fixation pathways in prokaryotes
Methane metabolism
Nitrogen metabolism
Oxidative phosphorylation
Carbon fixation in photosynthetic organisms
0.8 0.9 1.0 1.1 1.2 1.3
AAT/AA fold change

Figure S2

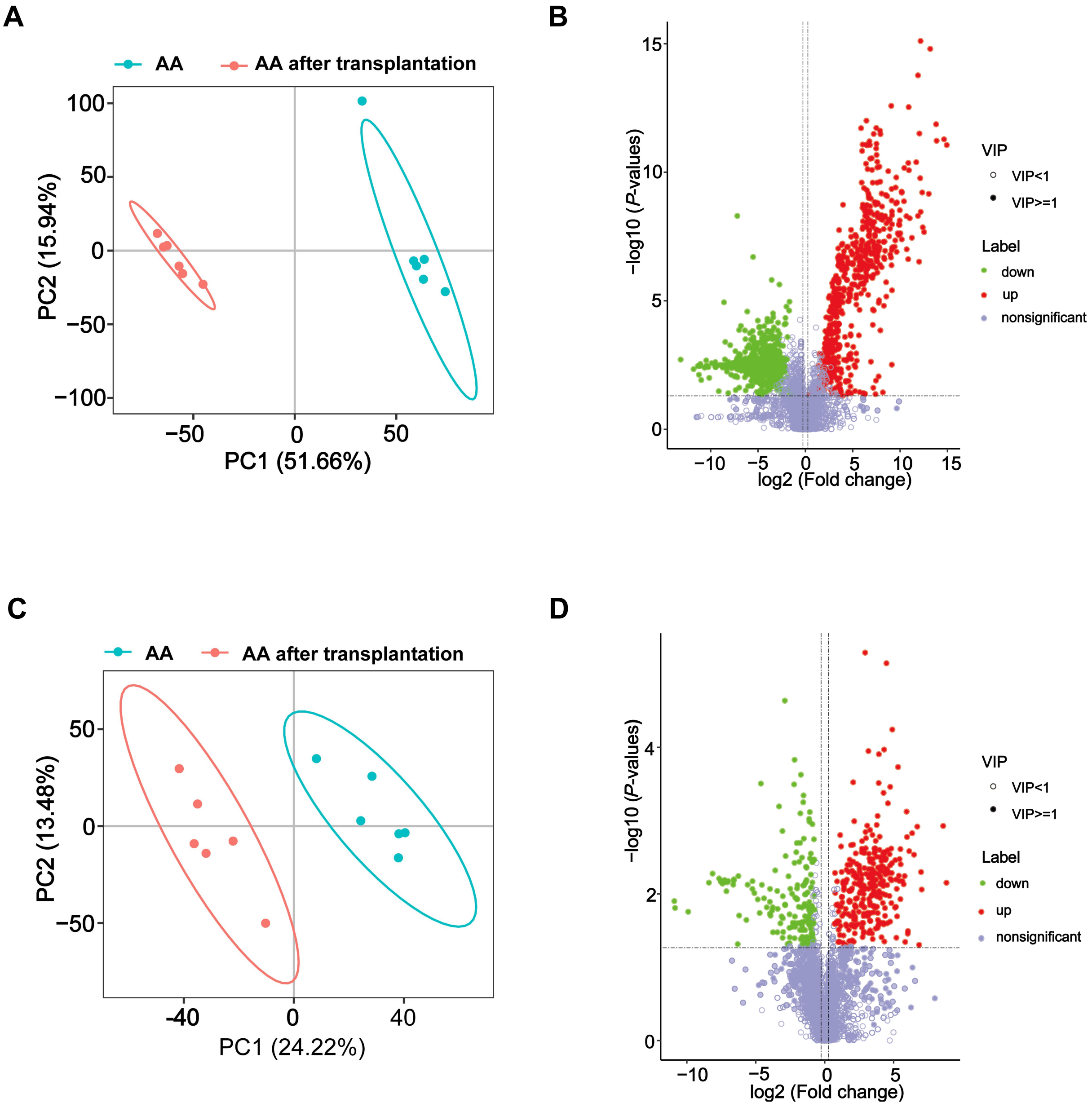
A
AA
AA after transplantation
PC2 (15.94%)
PC1 (51.66%)
100
50
0
−50
−100
−50
0
50
B
−log10 (P-values)
log2 (Fold change)
15
10
5
0
−10
−5
0
5
10
15
VIP
VIP<1
VIP>=1
Label
down
up
nonsignificant
C
AA
AA after transplantation
PC2 (13.48%)
PC1 (24.22%)
50
0
−50
-40
0
40
D
−log10 (P-values)
log2 (Fold change)
4
2
0
−10
−5
0
5
VIP
VIP<1
VIP>=1
Label
down
up
nonsignificant

Figure S3

A

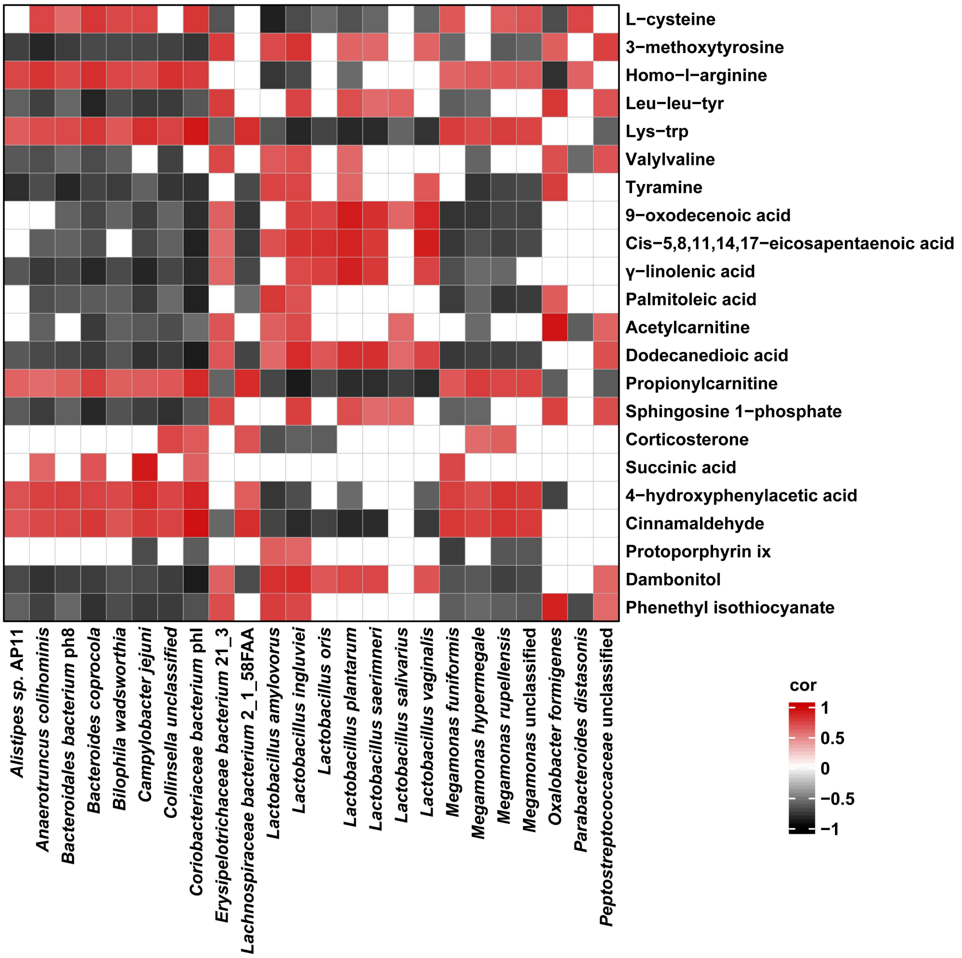
L−cysteine
3−methoxytyrosine
Homo−l−arginine
Leu−leu−tyr
Lys−trp
Valylvaline
Tyramine
9−oxodecenoic acid
Cis−5,8,11,14,17−eicosapentaenoic acid
γ−linolenic acid
Palmitoleic acid
Acetylcarnitine
Dodecanedioic acid
Propionylcarnitine
Sphingosine 1−phosphate
Corticosterone
Succinic acid
4−hydroxyphenylacetic acid
Cinnamaldehyde
Protoporphyrin ix
Dambonitol
Phenethyl isothiocyanate
Alistipes sp. AP11
Anaerotruncus colihominis
Bacteroidales bacterium ph8
Bacteroides coprocola
Bilophila wadsworthia
Campylobacter jejuni
Collinsella unclassified
Coriobacteriaceae bacterium phI
Erysipelotrichaceae bacterium 21_3
Lachnospiraceae bacterium 2_1_58FAA
Lactobacillus amylovorus
Lactobacillus ingluviei
Lactobacillus oris
Lactobacillus plantarum
Lactobacillus saerimneri
Lactobacillus salivarius
Lactobacillus vaginalis
Megamonas funiformis
Megamonas hypermegale
Megamonas rupellensis
Megamonas unclassified
Oxalobacter formigenes
Parabacteroides distasonis
Peptostreptococcaceae unclassified
cor
1
0.5
0
−0.5
−1

B

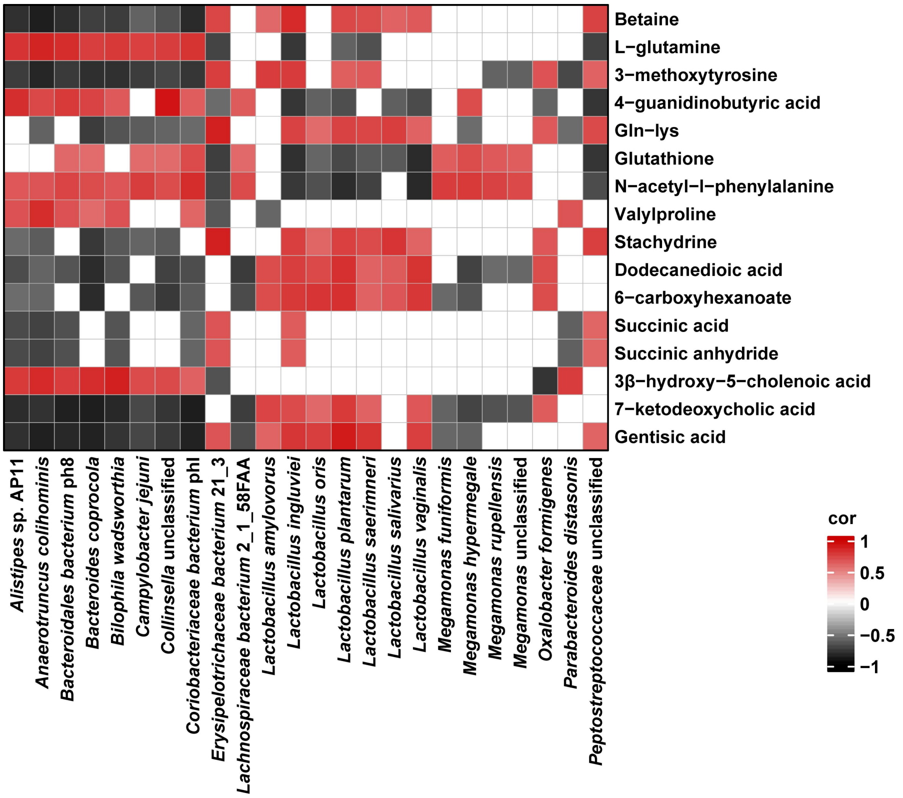
Betaine
L−glutamine
3−methoxytyrosine
4−guanidinobutyric acid
Gln−lys
Glutathione
N−acetyl−l−phenylalanine
Valylproline
Stachydrine
Dodecanedioic acid
6−carboxyhexanoate
Succinic acid
Succinic anhydride
3β−hydroxy−5−cholenoic acid
7−ketodeoxycholic acid
Gentisic acid
Alistipes sp. AP11
Anaerotruncus colihominis
Bacteroidales bacterium ph8
Bacteroides coprocola
Bilophila wadsworthia
Campylobacter jejuni
Collinsella unclassified
Coriobacteriaceae bacterium phI
Erysipelotrichaceae bacterium 21_3
Lachnospiraceae bacterium 2_1_58FAA
Lactobacillus amylovorus
Lactobacillus ingluviei
Lactobacillus oris
Lactobacillus plantarum
Lactobacillus saerimneri
Lactobacillus salivarius
Lactobacillus vaginalis
Megamonas funiformis
Megamonas hypermegale
Megamonas rupellensis
Megamonas unclassified
Oxalobacter formigenes
Parabacteroides distasonis
Peptostreptococcaceae unclassified
cor
1
0.5
0
−0.5
−1

Figure S4

A

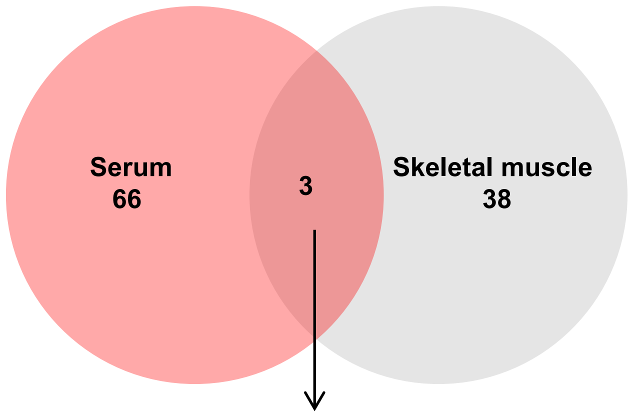

Common metabolites in serum and skeletal muscle

Metabolites	Category
3-methoxytyrosine	Amino acids, peptides, and analogues
Dodecanedioic acid	Fatty acyls
Succinic acid	Organic acids

B

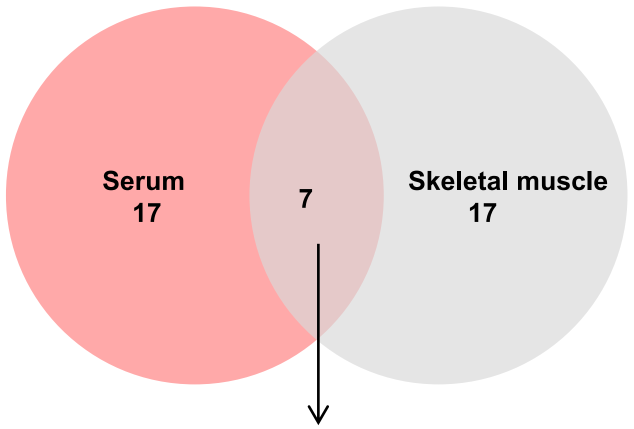

Common metabolic pathways in serum and skeletal muscle

KEGG level-3 metabolism pathways	KEGG level-2 metabolism pathways
Alanine, aspartate and glutamate metabolism	Amino acid metabolism
Glycine, serine and threonine metabolism	Amino acid metabolism
Tyrosine metabolism	Amino acid metabolism
Glutathione metabolism	Metabolism of other amino acids
Butanoate metabolism	Carbohydrate metabolism
Citrate cycle (TCA cycle)	Carbohydrate metabolism
Propanoate metabolism	Carbohydrate metabolism

Figure S5
