# Supplementary material for: Lactobacillus-derived protoporphyrin IX and SCFAs regulate the fiber size via glucose metabolism in the skeletal muscle of chickens
Source: mSystems. 2024 May 23;9(6):e00214-24. doi: 10.1128/msystems.00214-24 (PMC11237663; doi:10.1128/msystems.00214-24)
Supplement: Supplemental Material — Legends for supplemental figures and tables. [file msystems.00214-24-s0002.docx]

## SUPPLEMENTAL MATERIALS

**FIG S1**, PDF file, 1.07 MB.

**FIG S2**, PDF file, 1.09 MB.

**FIG S3**, PDF file, 1.16 MB.

**FIG S4**, PDF file, 1.70 MB.

**FIG S5**, PDF file, 57.0 KB.

**TABLE S1**, DOC file, 36.5 KB.

**TABLE S2**, XLS file, 60 KB.

**TABLE S3**, XLS file, 29.5 KB.

**TABLE S4**, XLS file, 26.0 KB.

**TABLE S5**, DOC file, 48.0 KB.

**TABLE S6**, DOC file, 38.5 KB.

**TABLE S7**, DOC file, 30.0 KB.

**FIG S1.** Microbial composition in the cecum of AA and AA after transplantation. (A) The composition of the cecal microbiota at the kingdom level. (B) Differential microbial genera in the cecum between AA and AA after transplantation. Only microbial genera with *P* < 0.05 are displayed. The data are the means ± SEM (n = 6 chickens).

**FIG** **S2.** AA after transplantation/AA fold change shows differences in KEGG level-3 microbial metabolic pathways between AA and AA after transplantation. (A) Amino acid metabolism. (B) Carbohydrate metabolism. (C) Lipid metabolism. (D) Metabolism of cofactors and vitamins. (E) Energy metabolism. **P* values < 0.05 were considered significant

**FIG S3.** The general metabolome profiles in the serum and pectoralis muscle of AA and AA after transplantation. (A) Serum metabolome profiles of AA and AA after transplantation based on the relative abundance visualized using a partial least square discriminant analysis (PLS-DA). (B) Volcano map of the differential serum metabolites between AA and AA after transplantation. (C) Pectoralis muscle metabolome profiles of AA and AA after transplantation based on the relative abundance visualized by a PLS-DA. (D) Volcano map of the differential pectoralis muscle metabolites between AA and AA after transplantation. In the volcano map, metabolites are shown as red (up), green (down), and purple (nonsignificant) dots. X-axis: AA after transplantation/AA fold change; Y-axis: -log10 *P* values.

**FIG S4.** Interactions among cecal microbiota, serum metabolites, and skeletal muscle metabolites. (A) Spearman’s rank correlations between differential cecal microbial species and differential FS-associated serum metabolites. (B) Spearman’s rank correlations between differential cecal microbial species and differential FS-associated skeletal muscle metabolites. Only strong correlations (R > 0.60 or R < -0.60, *P* < 0.05) are displayed in the heatmaps. The correlation scale ranged from -1 (black) to 1 (red).

**FIG S5.** Common FS-associated metabolite- and metabolite-enriched KEGG metabolic pathways between serum and skeletal muscle. (A) Significantly different metabolites shared in serum and pectoralis muscle between AA and AA after transplantation. (B) Significantly different metabolite-enriched pathways shared in serum and pectoralis muscle between AA and AA after transplantation.

**TABLE S1.** Summary of the sequence data generated from the cecum samples of AA and AA after transplantation

**TABLE S2.** Composition of cecal microbial metabolic pathways based on level 1-3 KEGG functions

**TABLE S3.** Fiber size (FS)-associated serum differential metabolites in serum

**TABLE S4.** Fiber size (FS)-associated differential metabolites in the pectoralis muscle

**TABLE S5.** The composition (as-fed basis, %) of the diets of Jingyuan chickens

**TABLE S6.** The composition (as-fed basis, %) of the diets of Arbor Acres chickens

**TABLE S7.** List of primer sequences
