# Supplementary material for: Lactobacillus-derived protoporphyrin IX and SCFAs regulate the fiber size via glucose metabolism in the skeletal muscle of chickens
Source: mSystems. 2024 May 23;9(6):e00214-24. doi: 10.1128/msystems.00214-24 (PMC11237663; doi:10.1128/msystems.00214-24)
Supplement: Table S1 — Summary of the sequence data generated from the cecum samples of AA and AA after transplantation. [file msystems.00214-24-s0003.doc]

Table S1 Summary of the sequence data generated from the cecum samples of AA and AA after transplantation

| Sample | Raw reads | Reads after QC | Clean reads | Contigs | N50(bp) | CDS |
| --- | --- | --- | --- | --- | --- | --- |
| AA 1 | 12351519600 | 11952732000 | 11631555300 | 298523 | 2032 | 705031 |
| AA 2 | 13459790400 | 12952175700 | 12552456600 | 487145 | 1924 | 772824 |
| AA 3 | 14986799100 | 14423040000 | 13831128000 | 436627 | 1566 | 854308 |
| AA 4 | 14234662500 | 13550953200 | 12885037800 | 422688 | 1669 | 843928 |
| AA 5 | 10823619600 | 10224408300 | 9532246500 | 374902 | 1328 | 867487 |
| AA 6 | 14986799100 | 14268020400 | 12823339200 | 473682 | 1929 | 228552 |
| AA after transplantation 1 | 15249725400 | 14270979600 | 13801577100 | 860098 | 1420 | 352516 |
| AA after transplantation 2 | 12260673600 | 11666710500 | 10205277300 | 527380 | 1755 | 485567 |
| AA after transplantation 3 | 12225967800 | 11646400200 | 9335821500 | 521276 | 1768 | 616814 |
| AA after transplantation 4 | 15249725400 | 14379202200 | 11417250600 | 629802 | 1394 | 497299 |
| AA after transplantation 5 | 14200460400 | 13534357500 | 9860722200 | 576599 | 1619 | 375782 |
| AA after transplantation 6 | 14723872800 | 14224164000 | 7166325000 | 347843 | 1729 | 249999 |
|  |  |  |  |  |  |  |
| Total | 164753615700 | 157093143600 | 135042737100 | 5956565 | 20133 | 6850107 |
| Average | 13729467975 | 13091095300 | 11253561425 | 496380 | 1678 | 570842 |
| SEM | 427140926 | 404065055 | 593551246 | 42966 | 65 | 68664 |
