# Supplementary material for: Lactobacillus-derived protoporphyrin IX and SCFAs regulate the fiber size via glucose metabolism in the skeletal muscle of chickens
Source: mSystems. 2024 May 23;9(6):e00214-24. doi: 10.1128/msystems.00214-24 (PMC11237663; doi:10.1128/msystems.00214-24)
Supplement: Table S5 — The composition of diets for Jingyuan chickens. [file msystems.00214-24-s0007.doc]

Table S5 The composition (as-fed basis, %) of diets for Jingyuan chickens

| **Ingredient (%)** | **0-6 weeks** | **7-12 weeks** | **> 13 weeks** |
| --- | --- | --- | --- |
| Ground corn | 58.43 | 62.56 | 66.05 |
| Rice bran meal | 4.00 | 6.18 | 5.3 |
| Soybean oil | 2.50 | 2.00 | 2.00 |
| Soybean meal | 28.08 | 21.72 | 20.88 |
| Rapeseed meal | 3.00 | 3.50 | 2.00 |
| Sodium chloride | 0.23 | 0.25 | 0.26 |
| Limestone | 1.38 | 1.14 | 1.28 |
| Ca_2_HPO_4_ | 1.11 | 1.28 | 0.92 |
| Lysine | 0.07 | 0.28 | 0.17 |
| Methionine | 0.17 | 0.03 | 0.07 |
| Threonine | 0.03 | 0.06 | 0.07 |
| *Premix | 1.00 | 1.00 | 1.00 |
| **Nutrition Level** |  |  |  |
| Metabolic energy (MJ/kg) | 12.01 | 11.97 | 12.16 |
| Crude protein (%) | 18.49 | 16.32 | 15.69 |
| Crude fat (%) | 5.15 | 4.73 | 4.80 |
| Crude fiber (%) | 3.25 | 3.16 | 2.92 |
| Crude ash (%) | 5.78 | 5.62 | 5.21 |
| Calcium (%) | 0.95 | 0.90 | 0.83 |
| Phosphorus (%) | 0.96 | 0.97 | 0.89 |
| Available phosphorous (%) | 0.36 | 0.39 | 0.31 |
| Lysine (%) | 1.06 | 1.13 | 0.98 |
| Methionine + Cysteine (%) | 0.78 | 0.62 | 0.61 |
| Threonine (%) | 0.79 | 0.70 | 0.67 |

* Premix contained per kg: vitamina A, 10,000 IU; vitamin D3, 2, 500 IU; vitamin E, 18.75 mg; vitamin K3 0.5 mg; vitamin B1, 2.5 mg, vitamin B2, 6.25 mg; vitamin B6, 2.5 mg; vitamin B12, 18.75 μg; nicotinic acid, 25.00 mg; pantothenic calcium, 12.50 mg; folic acid, 1.25 mg; biotin, 100μg; choline chloride, 800.0 mg; Fe, 78.00 mg (as iron sulfate monohydrate); Mn, 80.00 mg (as manganous oxide); Zn, 60.00 mg (as zinc sulfate); Cu, 8.00 mg (as copper sulfatepentahydrate); I, 0.40 mg (as calciumiodate); and Se, 0.20 mg (as sodium selenite).
