# Supplementary material for: Lactobacillus-derived protoporphyrin IX and SCFAs regulate the fiber size via glucose metabolism in the skeletal muscle of chickens
Source: mSystems. 2024 May 23;9(6):e00214-24. doi: 10.1128/msystems.00214-24 (PMC11237663; doi:10.1128/msystems.00214-24)
Supplement: Table S6 — The composition of the diets of Arbor Acres chickens. [file msystems.00214-24-s0008.doc]

Table S6 The composition (as-fed basis, %) of the diets of Arbor Acres chickens

| **Ingredient (%)** | **1-21 days** | **22-42 days** |
| --- | --- | --- |
| Ground corn | 58.50 | 61.10 |
| Wheat bran | 1.10 | 1.10 |
| Soybean meal | 31.50 | 28.00 |
| Soybean oil | 4.20 | 5.40 |
| Ca_2_HPO_4_ | 2.10 | 2.00 |
| Limestone | 1.30 | 1.10 |
| Sodium chloride | 0.20 | 0.20 |
| Lysine | 0.37 | 0.39 |
| Methionine | 0.20 | 0.18 |
| Threonine | 0.23 | 0.20 |
| *Premix | 0.30 | 0.30 |
| **Nutrition Level** |  |  |
| Metabolic energy (MJ/kg) | 12.54 | 12.97 |
| Crude protein (%) | 21.65 | 20.32 |
| Crude fat (%) | 6.58 | 7.80 |
| Crude fiber (%) | 2.42 | 2.25 |
| Crude ash (%) | 6.29 | 5.81 |
| Calcium (%) | 0.91 | 0.87 |
| Nonphytate phosphorus (%) | 0.49 | 0.47 |
| Sodium chloride (%) | 0.31 | 0.33 |
| Lysine (%) | 1.25 | 1.18 |
| Methionine (%)+Cysteine (%) | 0.93 | 0.87 |
| Threonine (%) | 0.50 | 0.46 |

* Premix contained per kg: vitamina A, 10,000 IU; vitamin D3, 2, 500 IU; vitamin E, 18.75 mg; vitamin K3 0.5 mg; vitamin B1, 2.5 mg, vitamin B2, 6.25 mg; vitamin B6, 2.5 mg; vitamin B12, 18.75 μg; nicotinic acid, 25.00 mg; pantothenic calcium, 12.50 mg; folic acid, 1.25 mg; biotin, 100μg; choline chloride, 800.0 mg; Fe, 78.00 mg (as iron sulfate monohydrate); Mn, 80.00 mg (as manganous oxide); Zn, 60.00 mg (as zinc sulfate); Cu, 8.00 mg (as copper sulfatepentahydrate); I, 0.40 mg (as calciumiodate); and Se, 0.20 mg (as sodium selenite).
