# Supplementary material for: Lactobacillus-derived protoporphyrin IX and SCFAs regulate the fiber size via glucose metabolism in the skeletal muscle of chickens
Source: mSystems. 2024 May 23;9(6):e00214-24. doi: 10.1128/msystems.00214-24 (PMC11237663; doi:10.1128/msystems.00214-24)
Supplement: Table S7 — Primer sequences. [file msystems.00214-24-s0009.doc]

Table S7 List of primer sequences

| Gene | Forward Primer | Reverse Primer |
| --- | --- | --- |
| *Acadl* | CTTTTGGGAAGACGGTTGCG | TGGAGATCAGACGCCCAGTA |
| *Acads* | GGCTGCTGGCTGTGGAAGTG | TTGACGCTGGCAATGACACCTG |
| *β-actin* | CACAGATCATGTTTGAGACCTT | CATCACAATACCAGTGGTACG |
| *CoX Va* | TTCAACAAACCAGACATCGATGCC | TTGGCAGAATGCTCCGGTAAC |
| *CoX VIIb* | AAATCTGGAATGGAGTGGGGC | AATGCCACAGCACAAGTGGTA |
| *Cpt1a* | CTGGGTTATTGCCACGAAGC | GCCATGGCTAAGGTTTTCGT |
| *Cyt c* | AGGAGGCAAGCACAAGACTG | TCCTCACCCCAAGTGATACC |
| *Bcat-1* | TCAGACCGCTTCAGAACCTCTCC | CGTGCCATCCTGTCCATGTTGAG |
| *Bckdk* | CCGACTTCGTGGGCATCATCTG | CTCGGCTGTGCTGAAGTGGTATTC |
| *LDH* | CTGTCTGGAGCGGAGTGAATGTTG | GTCCACCACCTGCTTGTGAACC |
| *IDH* | GTTATGGCTCTCTGGGCATGATGAC | GGTCTTGAGGCTAGTGTTGTTGTCC |
| *MB* | GAAAAGTGGAGGCCGACAT | TCAGATCTTCAGAGCCCTTCA |
| *MyHC SM* | AACGCCGCAACAACCT | TTCTTCTTCATCCGCTCC |
| *MyHC FRM* | GTTGACGTGCCTTCAGTACAGT | GCTTGTTCTGGGCCTCGATT |
| *MyHC FWM* | CCACCGATAGTGCCATTGACATCC | CCTGCTCTTCACGCTGCTTCTG |
| *MyoD* | CATCCGCTACATCGAGAGCC | TCGTAGCTGTTTCTCCTGCG |
| *MyoG* | AGCCAATGTGGCTGTTTCCA | ACACAGCGGTCAGTAGGAAAG |
| *PDH* | GGGAGGCTGGAGGTTCCTG | TGGTAACCACCGCTTTGTCT |
| *PFK* | TTCCCTCTTTTTCAGCCAAGA | CGAAGTAGACTTTGGCACCG |
| *PGC-1α* | CATGTGCAACCAGGACTCTGT | AGGCTCATTGCTGTACTGGC |
| *PK* | CGACTCCGAGCCAACCATTGC | GGTGCCGTGCGAGAAGTTGAG |
| *Tfam* | GAGAATTGACTGTGCTTGGAAAACCTAA | CATTAGCACAGCAGGATAAAGACATTACA |
| *SDH* | GAAGAGCACTGGAGGAAGCACAC | AGCGAATAGCAGGTGGAACACTTG |
